# Standard Anatomical and Visual Space for the Mouse Retina: Computational Reconstruction and Transformation of Flattened Retinae with the Retistruct Package

**DOI:** 10.1371/journal.pcbi.1002921

**Published:** 2013-02-28

**Authors:** David C. Sterratt, Daniel Lyngholm, David J. Willshaw, Ian D. Thompson

**Affiliations:** 1 Institute for Adaptive and Neural Computation, School of Informatics, University of Edinburgh, Edinburgh, Scotland, United Kingdom; 2 MRC Centre for Developmental Neurobiology, King's College London, London, United Kingdom; National Evolutionary Synthesis Center, United States of America

## Abstract

The concept of topographic mapping is central to the understanding of the visual system at many levels, from the developmental to the computational. It is important to be able to relate different coordinate systems, e.g. maps of the visual field and maps of the retina. Retinal maps are frequently based on flat-mount preparations. These use dissection and relaxing cuts to render the quasi-spherical retina into a 2D preparation. The variable nature of relaxing cuts and associated tears limits quantitative cross-animal comparisons. We present an algorithm, “Retistruct,” that reconstructs retinal flat-mounts by mapping them into a standard, spherical retinal space. This is achieved by: stitching the marked-up cuts of the flat-mount outline; dividing the stitched outline into a mesh whose vertices then are mapped onto a curtailed sphere; and finally moving the vertices so as to minimise a physically-inspired deformation energy function. Our validation studies indicate that the algorithm can estimate the position of a point on the intact adult retina to within 8° of arc (3.6% of nasotemporal axis). The coordinates in reconstructed retinae can be transformed to visuotopic coordinates. Retistruct is used to investigate the organisation of the adult mouse visual system. We orient the retina relative to the nictitating membrane and compare this to eye muscle insertions. To align the retinotopic and visuotopic coordinate systems in the mouse, we utilised the geometry of binocular vision. In standard retinal space, the composite decussation line for the uncrossed retinal projection is located 64° away from the retinal pole. Projecting anatomically defined uncrossed retinal projections into visual space gives binocular congruence if the optical axis of the mouse eye is oriented at 64° azimuth and 22° elevation, in concordance with previous results. Moreover, using these coordinates, the dorsoventral boundary for S-opsin expressing cones closely matches the horizontal meridian.

This is a *PLOS Computational Biology* Software Article

## Introduction

The retina projects directly and indirectly to a large number of areas in the central nervous system, such as the mammalian superior colliculus, lateral geniculate nucleus or visual cortex. Understanding the topographic mapping of these projections is a central feature of visual neuroscience [1]–[3]. However, there is considerable variation in the descriptions of the mappings. Anatomical studies tend to focus on *retinotopic* coordinates, examining the mapping of dorsal versus ventral (DV) retina and nasal versus temporal (NT) retina [4], [5]. Functional studies focus on *visuotopic* mappings: upper versus lower and central versus peripheral visual field [1], [6], [7]. The relation between retinotopic and visuotopic maps is simplest when the latter is centred on the optical axis of the eye but is more complicated for head-centred visuotopic coordinate systems, especially in laterally-eyed animals. Transformation between these coordinate systems is not intrinsically problematic but does require knowledge of where the optic axis projects in visual space. However, before reaching this stage there is a more fundamental problem: reconstructing the retina.

**Figure 1:**
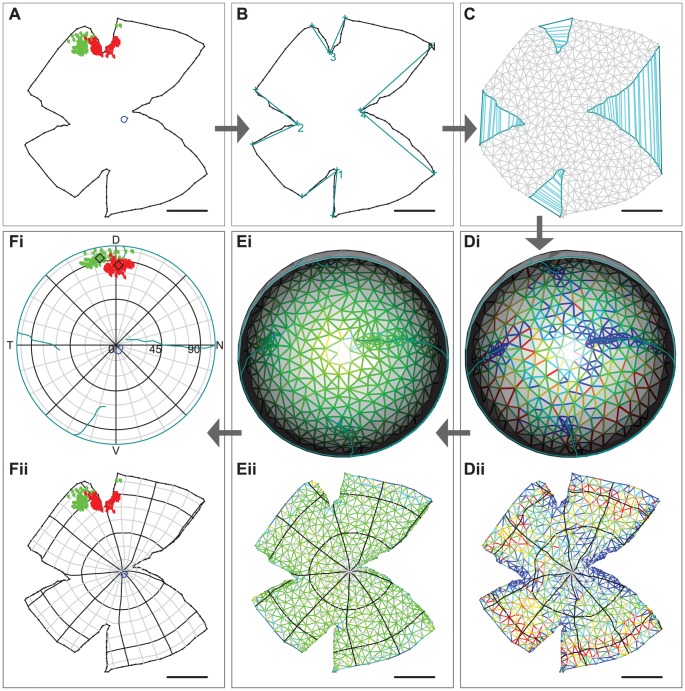
Overview of the method. A, The raw data: a retinal outline from an adult mouse (black), two types of data points (red and green circles) from paired injections into the superior colliculus and a landmark (blue line). **B,** Retinal outline with nasal pole (N) and cuts marked up. Each pair of dark cyan lines connects the vertices and apex of the four cuts. **C,** The outline after triangulation (shown by grey lines) and stitching, indicated by cyan lines between corresponding points on the cuts. **Di,** The initial projection of the triangulated and stitched outline onto a curtailed sphere. The strain of each edge is represented on a colour scale with blue indicating compression and red expansion. Cuts are shown in cyan. **Dii,** The strain plotted on the flat outline with lines of latitude and longitude superposed. **Ei,ii,** As Di,ii but after optimisation of the mapping. **Fi,** The data represented on a polar plot of the reconstructed retina. Mean locations of the different types of data points are indicated by diamonds. The nasal (N), dorsal (D), temporal (T) and ventral (V) poles are indicated. Cuts are shown in cyan. **Fii,** Data plotted on the flat representation, with lines of latitude and longitude superposed. All scale bars are 1 mm.

Historically, the anatomical organisation of the retina was frequently examined using serial sections, with the emphasis on example sections rather than reconstructions [8]. The introduction of retinal flat-mounts, also termed whole-mounts, [9], [10] was a major advance. Briefly, orienting marks are made in the retina whilst in the eye-cup, the retina is then dissected out and flattened with the help of a number of relaxing cuts. The flat-mount facilitated quantitative descriptions of the 2D distributions of different labels and markers across the retina. However, the relaxing cuts, together with tears that can occur during flattening, disturbs the retinal geometry significantly, which not only complicates comparison across retinae, but also can be problematic in interpreting results obtained from individual flat-mounted retinae. For example, various measures are used to quantify the regularity of mosaics of various cell types seen in flat-mounted retinae [11], [12], but these are susceptible to the existence of boundaries [13], both at the rim of the retina and those introduced by the relaxing cuts. In the study of topographic mapping, the locations of ganglion cells labelled by retrograde tracers injected into different locations in the target, the superior colliculus, have been compared in retinal flat-mounts [4], [5]. Foci of labelled cells can be separated, or even split, by relaxing cuts (see Figure 1A), complicating quantitative analyses.

We describe a method to infer where points on a flat-mount retina would lie in a standard, intact retinal space. The standard retina is approximated as a partial sphere, with positions identified using spherical coordinates. Our results show that the method can estimate the location of a point on a flat-mounted retina to within 8° of arc of its original location, or 3.6% of the NT or DV axis. This has allowed us to define a standard retinal space for the adult and for the developing mouse eye. Establishing the orientation of retinal space for the mouse, whose retina contains no intrinsic markers, requires experimental intervention. In the Results, we focus on data from adult mice. We show that a mark based on the centre of the nictitating membrane is reliable and this mark can be related to the insertion of the rectus eye muscles. Furthermore, we transform standard retinal space into visuotopic space and use the geometry of binocular vision, together with anatomical tracing, to address questions about the projection of the optical axis of the mouse eye into visual space [3], [14], [15].

Our Retistruct algorithm not only facilitates comparison of differential retinal distributions across animals but also allows analyses of distributions of labelled cells in spherical coordinates, obviating the distortions associated with 2D flat-mounts. Finally, transforming retinal coordinates into visual coordinates gives insights into the functional significance of retinal distributions.

## Design and Implementation

### The reconstruction algorithm

In this section we give an overview of the reconstruction algorithm; a more detailed description is contained in the Supplemental Materials and Methods (Text S1). The starting point of the algorithm is the flattened retinal outline (Figure 1A), which can include an image or labelled features. In Figure 1A the outline includes a landmark (blue line), in this case the optic disc, and the locations of retinal ganglion cells that have been retrogradely labelled following discrete injections of red and green fluorescent tracers into a retino-recipient central nucleus (the superior colliculus). One of the relaxing cuts has bisected the labelled foci – principally the red one. The first step is to mark the nasal retinal pole (in Figure 1B, “N”), which is defined by the perimeter of the long cut towards the optic disc from the peripheral fiducial mark based on the nictitating membrane (see Materials and Methods). The locations and extents of cuts and tears in the outline are also marked up (cuts 1–4 in Figure 1B). Because the retina in the eye-cup is more than hemispherical, the angle of the retinal margin (rim angle) measured from the pole of the retina is then supplied (Table 1 and see later section below). The outline is then divided into a mesh containing at least 500 triangles of roughly equal size, and the cuts and tears are stitched together (Figure 1C). This mesh is then projected onto a spherical surface, with all the points on the retinal margin lying on the circle defined by the rim angle (Figure 1Di). Each edge in the mesh is treated as a spring whose natural length is the length of the corresponding edge in the flat mesh. It is not possible to make this initial mapping onto the spherical surface optimal, so the springs are either compressed (blue), expanded (red) or retain their natural length (green). In the next step the springs are allowed to relax so as to minimise the total potential energy contained in all the springs, leading to the refined spherical mesh shown in Figure 1Ei.

**Table 1:** Measurements of mouse eyes at various stages of development.

Age	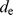 (µm)	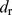 (µm)	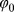 (°)
P0	1632±17	1308±17	127.13±1.92
P2	2146±13	1780±29	131.20±2.20
P4	2250±9	1857±20	130.58±1.40
P6	2450±41	1963±25	127.02±2.41
P8	2646±1	2088±34	125.31±1.81
P12	2786±15	2212±65	126.03±3.37
P16	2808±17	2043±17	117.10±0.96
P22	2958±35	2117±44	115.57±2.17
P64	3160±56	2161±30	111.56±1.89

The distance from the back of the eye to the surface of the cornea 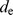, the perpendicular distance from the back of the eye to the rim of the retina 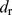 and the rim colatitude 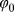 derived from this. Each measurement is averaged over the right and left eyes of two different animals, i.e. over four eyes in total.

The locations of mesh points in the flat-mount and their corresponding locations on the sphere define the relation between any point in the flat-mount and a standard spherical space. This relation is used to map the locations of data points and landmarks in the flat retina onto the standard retina. These can be visualised interactively on a 3D rendering of a sphere (see Figure 1D), or represented using a map projection such as the azimuthal equidistant projection centred on the retinal pole (Figure 1Fi). In all plots of retinal space we use the colatitude and longitude coordinate system, where colatitude is like latitude measured on the globe, except that zero is the pole rather than the equator. Map projections [16] such as that in Figure 1Fi are very useful for rendering retinal label into a standard retinal space. An alternative representation is shown in Figure 1Dii,Eii,Fii, where lines of latitude and longitude on the standard spherical retina have been projected onto the flat structure. The jump between Figure 1Dii and 1Eii illustrates the improvement in the appearance of the mapping achieved after minimising the energy.

In order to analyse data points on the standard retina, we used spherical statistics [17]. The mean locations of groups of data points on the sphere (diamonds in Figure 1Fi) are computed using the Karcher mean and we used density estimation to produce contour plots (see Supplemental Materials and Methods, Text S1).

### Performance of the algorithm

**Figure 2:**
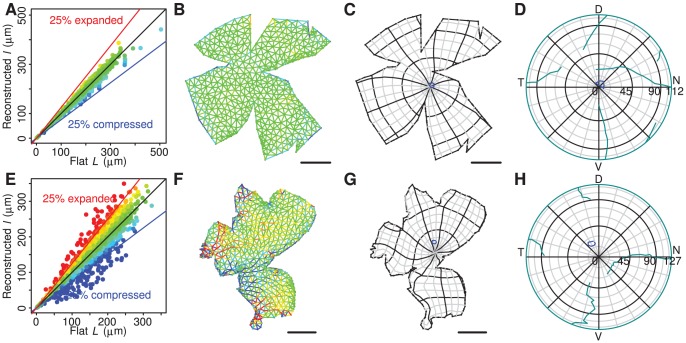
Examples of reconstructed retinae. A–D, An example of a reconstruction of an adult retina with low deformation measure 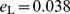. **A,** Plot of length of edge on the sphere versus length of edge on the flat retina. Red indicates an edge that has expanded and blue a edge that has been compressed. **B,** The log strain 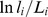 indicated using the same colour scheme on the flat retina. **C,** The flat representation of lines of latitude and longitude with the optic disc (blue). **D,** The azimuthal equidistant (polar) representation showing the locations of the cuts and tears (cyan) and the location of the optic disc (blue). **E–H,** An example of a reconstruction of a P0 retina with high deformation energy 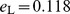. Meaning of E–H same as for corresponding panel in A–D. All scale bars are 1 mm.

To assess the amount of residual deformation at the end of the energy minimisation procedure, we plotted the length of each edge 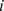 in the spherical mesh 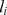 versus the length of the corresponding edge in the flat mesh 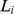 (Figure 2A), using the same colour scale as in Figure 1D,E. A measure of the overall deformation of reconstruction is:
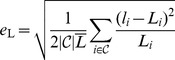
(1)where the summation is over 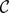, the set of edges, the mean length of an edge in the flat mesh is 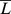, and the number of edges is 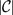. Physically, this measure is the square root of the elastic energy contained in the notional springs. It is constructed so as to be of a similar order to the mean fractional deformation.

We used our algorithm on 297 flat-mounted retinae, 288 of which could be reconstructed successfully, 7 of which failed due to, as-yet unresolved, software problems and 2 of which were rejected because of unsatisfactory reconstructions (see below). Figure 2A shows the reconstruction with the lowest deformation measure 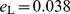 and Figure 2E the example with a high value of 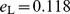. The arrangement of the grid lines in the example with lower deformation looks qualitatively smoother and more even than in the example with higher deformation measure (Figure 2C,G). The strain plot for the retina with the lower deformation (Figure 2B) indicates that almost all edges are unstressed, whereas in the retina with higher deformation (Figure 2F) there are many more compressed and expanded edges. It can be seen that the retina in Figure 2E–H has a much less distinct margin than in Figure 2A–D. This makes it harder for the algorithm to create an even mapping, as local roughness in the rim forces significant deformation of the surrounding virtual tissue when morphed onto the sphere.

**Figure 3:**
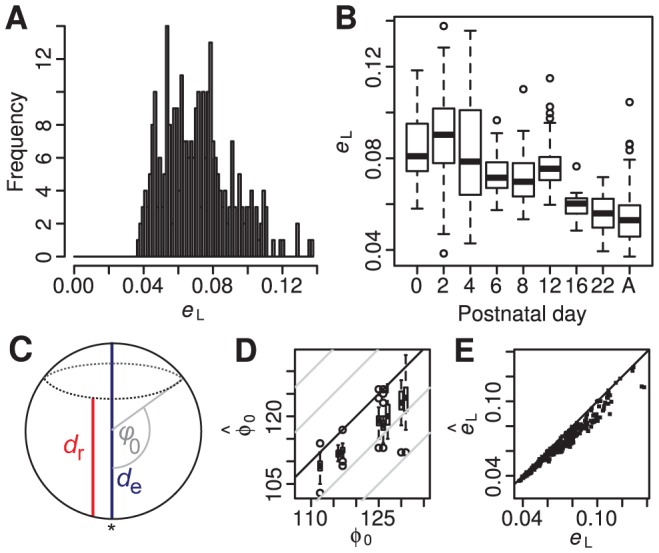
Deformation of reconstructions and the effect of rim angle. A, Histogram of the reconstruction error measure 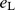 obtained from 288 successfully reconstructed retinae. **B,** Relationship between deformation measure and age. “A” indicates adult animals. **C,** Schematic diagram of eye, indicating the measurements 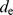 and 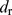 made on mouse eyes at different stages of development, and the rim angle 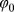 derived from these measurements. Note that rim angle is measured from the optic pole (*). **D,** Rim colatitude 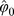 that minimises reconstruction error versus the rim angle 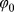 determined from eye measurements. Solid line shows equality and grey lines indicate ±10° and ±20° from equality. **E,** Minimum reconstruction error 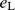 obtained by optimising rim angle versus reconstruction error 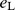 obtained when using the rim angle from eye measurements. Solid line indicates equality.

Thus the deformation measure 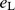 gives some indication of the apparent quality of the reconstruction. A value greater than 0.2 indicates a problem with the stitching part of the algorithm; the 2 such reconstructions were rejected and are not included in the 288 successful reconstructions. Noticeably bad reconstructions tend to have 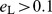. We recommend checking the mark-up of cuts and tears in any retinae with 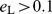. The mean deformation measure was 0.071, the median was 0.070 (Figure 3A), and 27 out of 288 retinae had a deformation measure exceeding 0.1, including the retina illustrated in Figure 2E–H as an, intentionally, poor dissection. With 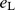 = 0.118, it falls above the 97.5th percentile of the retinae illustrated in Figure 3A. The larger deformations tend to come from younger animals (Figure 3B), reflecting the difficulty of dissecting retinae out of these animals cleanly due to the more delicate nature of younger tissue.

It should be noted that retinae which have lost tissue due to poor dissection can be forced onto the spherical surface by the algorithm, albeit with high 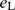 values. Reconstruction of such retinae should not be attempted, since remaining tissue will be mapped to inappropriate regions of the sphere.

### Determination of the rim angle

To determine the rim angle of mouse eyes at varying stages of development we measured the distance 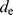 from the back of the eye to the front of the cornea and the distance 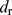 from the back of the eye to the edge of the retina (Figure 3C). We then computed the colatitude (the angle measured from the retinal pole) 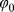 of the rim using the formula:
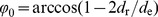
(2)The measurements and derived latitudes for animals of various ages are shown in Table 1.

An alternative approach to setting the rim angle is to infer, for each individual retina, the rim angle that minimises the deformation. This was done by repeating the minimisation for rim angles at 1° intervals within a range 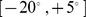 of the rim angle determined by measurement as above. A comparison of the measured and inferred rim angles is shown in Figure 3D. It can be seen that inferred rim angle is usually less than that obtained from measurements of standard retinae. Figure 3E shows a comparison of the reconstruction error obtained using the measured and inferred rim angles. The maximum decrease in the reconstruction error is 19.1%, with the mean improvement being 7.2%. We concluded that this improvement was not sufficiently great to add automatic refinement of the rim angle to the algorithm.

### Estimate of errors of the reconstruction algorithm from optic disc location

**Figure 4:**
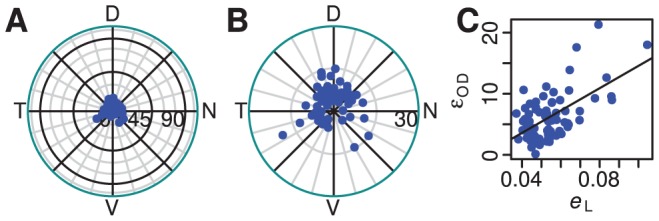
Estimation of reconstruction error using optic disc location. A, Inferred positions of optic discs from 72 adult reconstructed retinae plotted on the same polar projection. The colatitude and longitude of the Karcher mean is (3.7°, 95.4°). The standard deviation in the angular displacement from the mean is 7.4°. **B,** The same data plotted on a larger scale. **C,** The relationship between the deformation of the reconstruction and distance 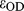 of the inferred optic disc from the population mean. There was a significant correlation between the two (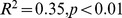).

The deformation measure gives an indication of how easy it is to morph any particular flattened retina onto a partial sphere, but it does not indicate the error involved in the reconstruction, i.e. the difference between the inferred position on the spherical retina and the original position on the spherical retina. The ideal method for estimating the error would be to flatten a retina marked in known locations, and then compare the inferred with the known locations. However, this proved to be technically very difficult and so we tried another method of estimating the error that uses the inferred locations of the optic discs across a number of retinae. In mice, the optic disc is located “rather precisely in the geometric center of the retina” [18], though this has not, as far as we are aware, been measured. We marked the optic disc in 72 flat-mounted adult retinae, and the distribution of the centres of the inferred locations of these optic discs is shown in Figure 4A,B. The mean colatitude and longitude of these optic discs is (3.7°, 95.4°) with a standard deviation of 7.4°. The mean is therefore 3.7° away from the geometrical centre of the retina, in good agreement with the qualitative observation that the optic disc is at the geometric centre of the retina. Under the, questionable, assumption that none of the variability is biological, this suggests that an upper bound on the accuracy of the reconstruction algorithm is 7.4°. There is a significant relationship between the deformation error 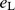 and the inferred distance of the optic disc from the mean optic disc location (Figure 4C). If reconstructions require accuracy to less than 7.4°, this could be achieved by increasing the stringency of 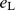 values used to reject reconstructions. Rounding up this error gives a value of 8°, which is 3.6% of the 223° along the nasotemporal axis of the adult eye. It is worth noting that the error of reconstruction depends not only on the algorithm, but also the data presented to it, which is intrinsically variable.

## Results

### Distributions of ipsilateral and contralateral retinal projections

We now describe the first application of the reconstruction algorithm. The relationship between the projections from the mouse retina to the ipsilateral and contralateral dLGN has been studied in retina flat-mounts following injection of retrograde tracer into the dLGN [3]. Reconstructing the retinae of individual animals that have had retrograde tracer injected into primary visual areas enables comparisons of projection patterns across animals independent of distortions introduced by retinal dissection. Moreover, having a standard retinal space means that data from multiple animals can be used to create aggregate topographic maps.

**Figure 5:**
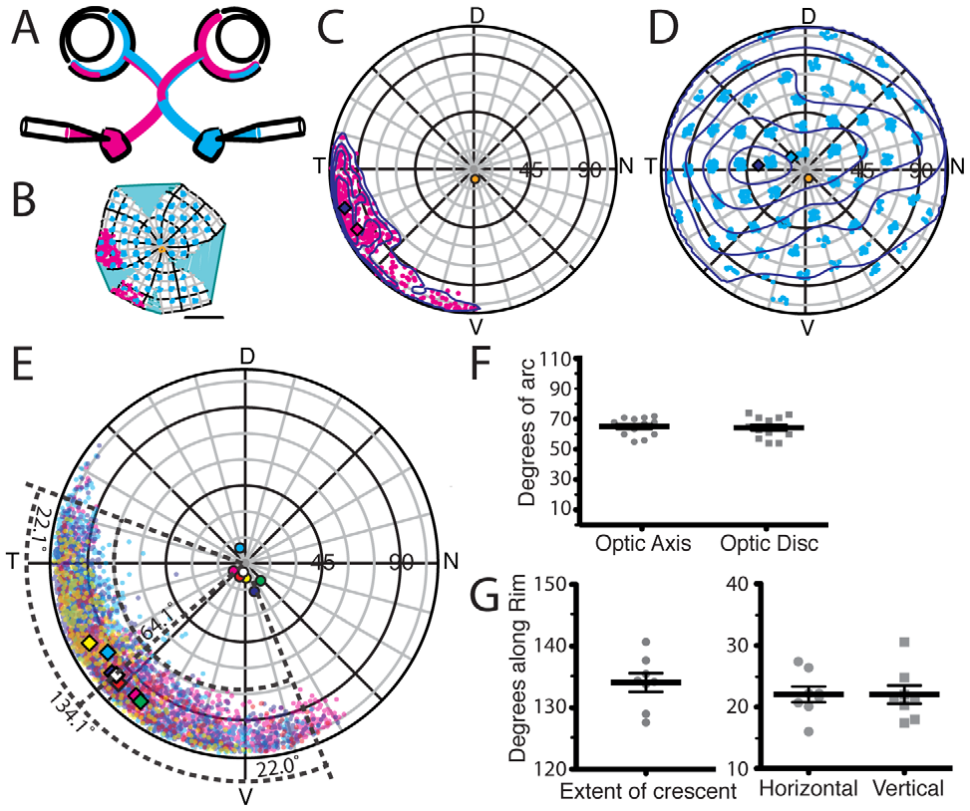
Measurement of the ipsilateral projection. A, Schematic illustrating the retinal label resulting from bilateral injections of Fluoro-Ruby (magenta) and Fluoro-Emerald (cyan) dye into the dLGN. **B,** Flat-mounted retina with label resulting from bilateral injections of Fluoro-Ruby (magenta) and Fluoro-Emerald (cyan) into left and right dLGN, respectively. **C–D,** Azimuthal equilateral projection of reconstructed retina in B. Isodensity contours for 5%, 25%, 50%, 75% & 95% are plotted using the kernel density estimates (KDEs) for fully sampled retinae (C) or kernel regression (KR) estimates for partially sampled retinae (D). Blue diamond is the peak density and magenta (C) or cyan (D) diamond is the Karcher mean. Yellow circle is the optic disc. **E,** Composite plot with ipsilateral label from unilateral injections (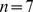). Black dashed lines represent the median angle from the optic axis to the peripheral edges of the 5% isodensity contour. Coloured diamonds represent the Karcher means of the label in the individual retinae and large coloured circles are the optic discs for the individual retinae. White square and circle represent the average Karcher mean. The central dashed angle represents the median central edge of the 5% isodensity contour. **F,** Mean distances from either optic disc or optic axis of reconstructed retinae to the central edge of the 5% isodensity contour along a line passing through the Karcher mean of the label. **G,** The extent of the ipsilateral label and the distance beyond the horizontal and vertical axes. Grid spacing is 20°. In F–G, line represents the mean and error bars are standard error of the mean. Scale bar in C & D is 1 mm.

The reconstruction method has enabled the quantification of the binocular projection from the retina to the dLGN across multiple animals. To label the projection, the dLGN in adult mice was injected either with Fluoro-Ruby or Fluoro-Emerald. Further, in some animals, the injections were bilateral (see Figure 5A and Supplemental Materials and Methods, Text S1, for details). The Retistruct program was used to reconstruct the retinae. The plots in Figures 5B–D show the label in *one* retina from an animal that had received *bilateral* injections into the dLGN (Figure 5A). These plots were done using an in-house camera-lucida set-up, sampling the entire ventrotemporal crescent for the ipsilateral retina and one in nine 150 m square boxes for the contralateral label (Figure 5B). The ipsilateral projection (Figure 5C) is restricted to the ventrotemporal crescent whereas label from the contralateral projection (Figure 5D) is distributed widely. The nature of the overlap between the uncrossed and crossed projections is evident in Figure 5B. Having reconstructed the retinae into a standard space, we quantified the projection patterns by deriving kernel density estimates (KDEs) of the underlying probability of data points appearing at any point in the retina and represented these estimates using contours that exclude 5%, 25%, 50% and 95% of the points (Figure 5C). In the case of the contralateral label, the data consisted of cell counts within defined boxes on the flattened retina. Here we used kernel regression (KR) as the source for the contouring algorithm (Figure 5D; see Supplemental Materials and Methods, Text S1, for details). The Karcher mean of the data points is represented by the magenta and cyan diamonds in either plot and the peak density for the kernel is represented by the blue diamond. It is worth noting that these two measures often give different locations, as would be expected from skewed distributions.

**Figure 6:**
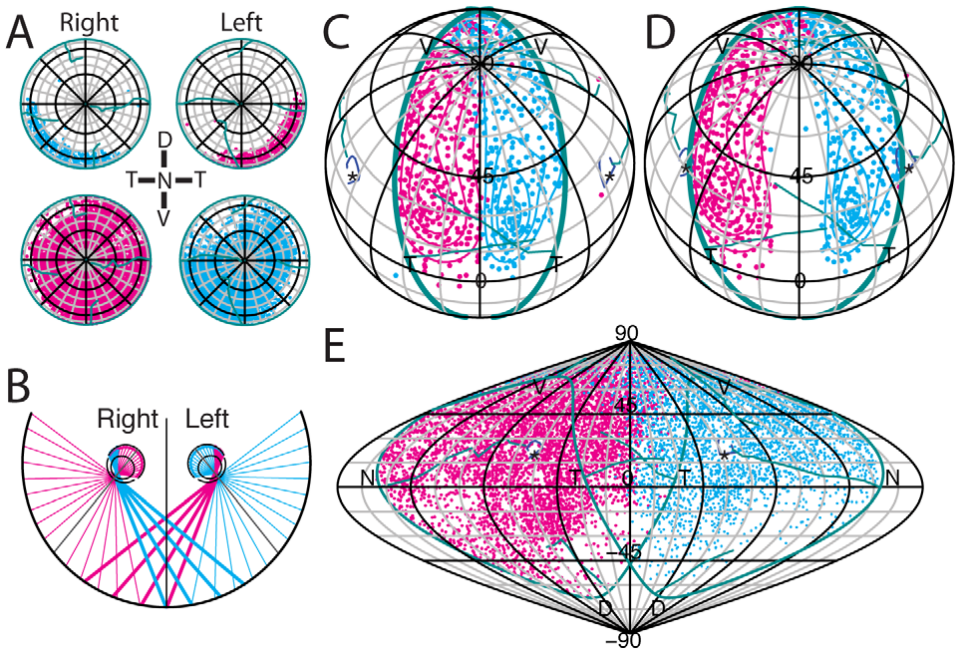
Alignment of the binocular zone in visuotopic coordinates. A, Azimuthal equilateral projections in standard *retinal* space of left and right retinae with ipsilateral (upper) and contralateral (lower) label resulting from bilateral injections of Fluoro-Ruby (magenta) and Fluoro-Emerald (cyan) into left and right dLGN, respectively, of the same mouse. Plots were generated from stitched 10× epifluorescent images and cell locations detected using ImageJ. For this figure, we have abandoned the convention of always plotting nasal retina to the right. **B,** Schematic illustrating the approximate projection of *retinal* space onto *visual* space. When the orientation of the optic axis (grey line) is optimal, the ipsilateral crescent is projected entirely to the opposite visual field. Note that due to the refraction in the lens the visual field is estimated to be 180° for each eye. **C–D,** Orthographic projections in central *visuotopic* space of the two ipsilateral retinae in A with optic axis (*) at 64° azimuth; 22° elevation (C) and with optic disc at 60° azimuth; 35° elevation (D). **E,** Sinusoidal projection of contralateral retinae in B with the optic axis (*) at 64° azimuth; 22° elevation. Labels N, D, T, V indicate the projection of the corresponding pole of the retina. Grid spacing is 15°.

Obtaining uniform and complete injections of tracer into the dLGN can be difficult and can result in variability in the pattern of label (e.g. the contralateral retinae in Figure 6A). We have taken advantage of standard retinal space to measure the extent of the ipsilateral projection by making a composite plot of data from 7 different animals (Figure 5E), which shows that the average ipsilateral projection occupies a crescent in ventrotemporal retina. The decussation line for the aggregate ipsilateral population is 64.1±1.6° from the retinal pole, which in these 7 animals is very close to the optic disc. The distance from the optic disc to the decussation line is 63.4±1.3° (Figure 5F). The ipsilateral crescent spans an average of 134.1±1.5° of the rim extending from 22.1±1.3° beyond the temporal pole to 22.0±1.5° beyond the ventral pole (Figure 5G).

### Transformation to visuotopic coordinates

The geometry of binocular vision implies that the ipsilateral decussation line should correspond to the vertical meridian in the adult mouse's visual field [3], [6]. In order to investigate this prediction, we sought to map the retina onto visual space. The mapping of visual space onto the retina is determined by the orientation of the optic axis and the optics of the eye. We assume that the optic axis corresponds to the retinal pole of the spherical retinal coordinate system. Thus the optic axis is close to but not coincident with the optic disc. The location of the optic *disc* has been estimated to be projected to a point 60° lateral to the vertical and 35° above the horizontal meridian in anaesthetised mice [14]. Alternatively, the optic *axis* has been reported to be 64° lateral to the vertical and 22° above the horizontal meridian in ambulatory mice [15]. In anaesthetised mice, Dräger and Hubel noted that the eyes were always diverted outwards [1].

In principle, the deviation of a ray by the eye can be estimated by means of a schematic model of the mouse eye [19], [20]. We investigated using one such model [20] to determine the deviation of paraxial rays. The schematic eye model is not, however, constructed to account for wide-field rays, so we decided to approximate the effect of the optics of the eye by making the deviation of a rays passing through the posterior nodal point of the eye, which is approximately the centre of the eye, proportional to the ray's angle of incidence. The constant of proportionality is such that rays at 90 to the eye will be projected onto the edge of the retina, regardless of the retina's rim angle (see Figure 6B). The mapping of the eye onto visual space is effected by a coordinate transformation in which first the approximate optics are used to project points on the retina through the centre of the eye onto a notional large concentric sphere about the eye representing visual space. Then the locations of the points on this “celestial” sphere are measured in terms of elevation, the angle above the horizontal and azimuth, the angle made between the point's meridian plane and the zero meridian plane, i.e. the vertical plane containing the long axis of the mouse. By convention [3], [6], [14], projections of visual space are presented as though the mouse is sitting facing the observer, so that the azimuth angle is positive in the left visual field.

Using the above conventions to test whether the position of the ipsilateral decussation line corresponds to the vertical meridian in visuotopic space, we have transformed the retinotopic location of ipsilateral retinal ganglion cells, following bilateral injections, into head-centred visuotopic space [21]. To minimise between-animal variability, we used bilateral injections into the dLGN: injecting Fluoro-ruby on one side and Fluoro-emerald on the other side (see Figure 5A). Figure 6A illustrates the distribution of retrogradely-labelled ganglion cells in the ipsilateral (upper plots) and contralateral (lower plots) retinae. For this Figure, we have abandoned the standard representation of the nasal retina to the right in order to emphasise the mirror-symmetry of the projections. Injection of Fluoro-emerald into the right dLGN leads to label restricted to the ventrotemporal crescent in the right retina but widespread labelling in the left retina; a complementary pattern is seen for an injection of Fluoro-ruby into the left dLGN. When the retinal distribution of the ipsilateral ventrotemporal crescent neurons is transformed into visuotopic space using the optic axis coordinates of 64° azimuth and 22° elevation [15], and plotted in an orthographic projection of the central visual field, the decussation line lines up with the vertical meridian. These plots have been rotated with 50° elevation to include the upper part of the visual field beyond 180° (Figure 6C). If, in contrast, the ipsilateral projection is transformed using the optic disc coordinates of 60° azimuth and 35° elevation [14], there is an evident visual mismatch between the two decussation lines (Figure 6D). To examine the visuotopic extent of the contralateral projection in the two eyes, we displayed the data in a sinusoidal map projection to include the full visual field through both eyes (Figure 6E). This demonstrates that the decussation pattern in the contralateral projection is not as sharp as that in in the ipsilateral projection. Intriguingly, it appears that inferior-central field, a region that would be shadowed by the nose, is also under-represented.

Given these observations and our group data for optic disc location (Figure 5E), this indicates a revised optic disc projection of 66° azimuth and 25° elevation. Having fixed the location of the eye in visual space, our data on the uncrossed projection predict that the width of binocular visual field is 52° at its greatest width, slightly larger than the 30–40° of Dräger and Hubel [1], but within the range of 50–60° estimated by Rice et al. [22] and Coleman et al. [3]. The binocular field starts a few degrees below the horizon and continues well behind the animal's head – very like the situation in the rabbit [23].

### Eye muscles and S-opsin in retinal and visuotopic coordinates

**Figure 7:**
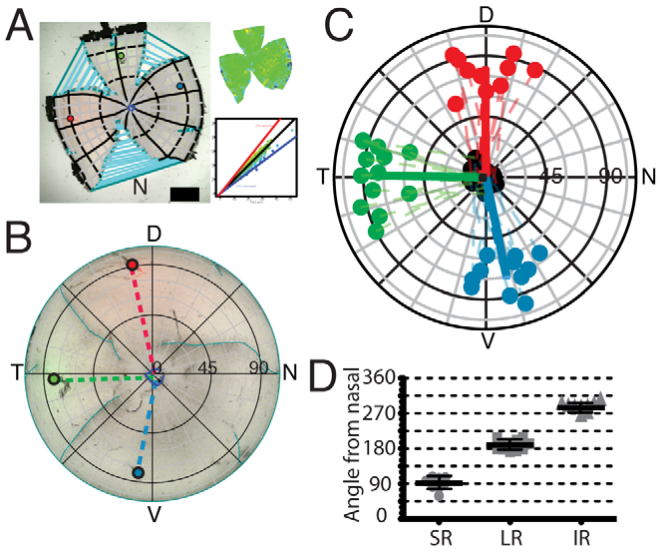
Measurement of muscle insertion angles. A, Flat-mounted retina showing stitching and insertions for superior rectus (red), lateral rectus (green) and inferior rectus (blue). N indicates nasal cut. Plots on right represent the distortions introduced by reconstructing retina (see Figure 2 for explanation). **B,** Azimuthal equilateral projection of reconstructed retina in A. Dashed lines represent vectors connecting muscle insertion point to the optic disc. **C,** Muscle insertion points from 17 retinae. Solid black circles represent the optic discs for individual retinae. Dashed lines represent the line from each individual insertion point to its respective optic disc. Solid lines are from the Karcher mean insertion to the Karcher mean location of the optic disc. Grid Spacing is 15°. **D,** Plot of the angles of the angles of vectors connecting muscle insertions of Superior Rectus (SR), Lateral Rectus (LR) and Inferior Rectus (IR) to the individual optic discs. Bar represents the mean and error-bars are standard deviation.

To examine the orientation of the eye, the locations of the insertions of superior, lateral and inferior rectus into the globe of the eye were marked onto the retina (Figure 7A; see Supplemental Materials and Methods, Text S1, for procedure). The nasal pole of the retinae is determined with reference to the nictitating membrane. The retinae were reconstructed and plotted in an azimuthal equidistant polar projection and the vectors connecting the insertion points and the optic disc were plotted (Figure 7B). Once in a standard space, the muscle insertion points (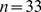) from all the retinae (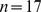) were plotted in the same plot and the vectors connecting the Karcher mean of each muscle insertion and the Karcher mean for the optic disc location were plotted (Figure 7C). Figure 7D shows the mean vector angles: lateral rectus, at 184.9±3.6°, is directly opposite the nasal cut, superior rectus is at 91.3±5.9° and inferior rectus is at 284.2±4.1°,where nasal is 0°. It is noticeable that there is considerable variability in the locations of the muscle insertions, certainly when compared to the variability of the optic discs. A considerable contributory factor in this is the large extent of the muscle and the relative difficulty in determining the centre of the muscle.

**Figure 8:**
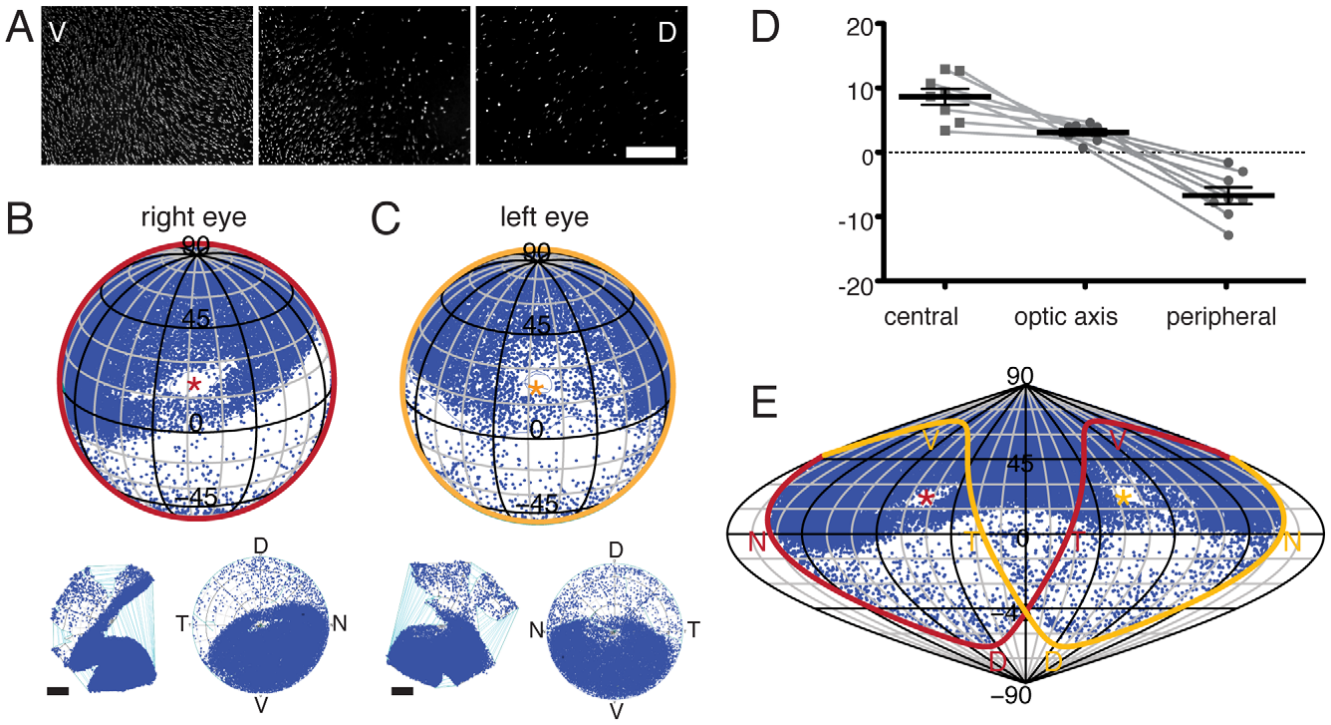
Visuotopic axes with respect to S-opsin distribution. A, S-opsin staining in dorsal, central and ventral retina. Images acquired at 20× magnification. Scale bar is 100 m. **B–C,** S-opsin distribution for right (A) and left (B) eyes plotted in orthographic projection centred on optic axis (*) at 22° elevation and 64° azimuth. Bottom left plot is flat-mounted retina. Bottom right plot is azimuthal equilateral plot. Plots were generated from stitched 10× epifluorescent images and cell locations detected using ImageJ [29]. There are slight differences between the two eyes in the exact angle of density transition with respect to the horizontal meridian and in the density of staining around the optic disc. These will reflect experimental variance. Again our normal convention of showing nasal to the left has been relaxed. Scale bar is 1 mm. **D,** The average offset of the S-opsin density-transition from the horizontal meridian in central and peripheral visual field (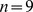). Error bars are SEM. **E,** S-opsin distribution for both eyes plotted in a sinusoidal projection with same optic axis (*) as in C. Yellow outline is edge of left retina; red outline is edge of right retina. Labels N, D, T, V indicate the projection of the corresponding pole of the retina. Grid spacing is 15°.

The location of the optic axis at 64° azimuth and 22° elevation [15] determines the location of the vertical and horizontal meridians on the eye. The location of the ipsilateral decussation line confirms this azimuthal value (Figures 5E & 6C). As the mouse retina has no pronounced horizontal streak, we looked at the distribution of short wavelength opsin (S-opsin) in the retina. Haverkamp et al. [24] describe a very distinct distribution pattern in the retina, with high density ventral, low density dorsal and an abrupt transition and suggested that the transition coincided with the horizontal meridian. Figure 8A shows immuno-staining for S-opsin from dorsal, central and ventral retina. The density difference between dorsal and ventral is marked and the transition is abrupt. Figure 8B & 8C illustrate the transformation of the S-opsin distribution from retinal flat-mounts to standard retinal space to an orthographic representation of visuotopic space centred on the optic axis. In these plots, the transition is 3.3±0.3° above the horizontal meridian at the level of the optic axis and is tilted by 13.8±3.6°, so that transition is 7.8±2.0° above the horizontal meridian in central visual field and 6.0±1.6° below the horizontal meridian peripherally (Figure 8D). The label from both left and right retinae was also plotted in a sinusoidal projection (Figure 8D) to illustrate that the rotation seen in the orthogonal plots for each eye is symmetric along the vertical meridian of the entire visual field (Figure 8E).

In summary, with the location of the optic axis determined by the optimal match of the decussation line with the vertical meridian, the change in S-opsin staining nicely coincides with the horizontal meridian, at least for the central visual field, as predicted by Szél et al. [25]. It is worth noting that S-opsin is mostly co-expressed with medium wavelength opsin (M-opsin) [24], [26], and that this would make these cones in upper visual field respond to a broader spectrum, which may have implications for the ability to detect a larger range of objects above the mouse. The precise distribution of S-opsin is not uniformly agreed upon [24]–[26]. However, when plotting the distributions from Szél et al [25] to visuotopic space using Retistruct (data not shown), we get a very similar distribution to that seen in Figure 8B–C.

## Availability and Future Directions

The reconstruction and transformation methods described here have been implemented in R [27] and use the Triangle library [28] for mesh generation. The retinae in this paper were reconstructed and analysed using version 0.5.7 of the Retistruct package (Dataset S1). The package, including full installation instructions, is also available anonymously and for free under the GPL license from http://retistruct.r-forge.r-project.org/. The package has been tested with R version 2.15.2 on GNU/Linux (Ubuntu 12.04), MacOS X 10.8 and Microsoft Windows Vista. The user guide, available from the same site, contains details of the two main data formats Retistruct can process. These are either in the form of coordinates of data points and retinal outline from an in-house camera-lucida setup, or in the form of bitmap images with an outline marked up in ImageJ ROI format [29]. There is a GUI interface to facilitate the marking-up of retinae and displaying reconstructed retinae.

The program could be applied to flat-mount preparations of retinae from any vertebrate species at any age, provided the globe of the eye is approximately spherical, and it would be possible to add extra analysis routines. When examining retinae with mosaic labelling [30], reconstructing the retina into its original spherical coordinates would make it possible to determine more accurately the relative distances between cells between peripheral and central retina. To implement mosaic analysis would require computation of a Voronoi tessellation on the sphere, which could be implemented by doing the Voronoi tessellation on a conformal (Wulff) projection [31]. Further visualisation methods could also be added. Using equations similar to those in the literature [21], locations on the retina could be projected onto a screen placed orthogonal to the optic axis, thus enabling a direct translation of visual stimulus space onto retinal coordinates. It would also be possible to map visual space onto retinal coordinates, by inverting the operations to map the retina onto visual space.

It should be stressed that the current version is not intended for use on partial retinae. However, it might eventually be possible to locate incomplete retinae in standard retinal space, if there enough markers to orient the partial retina correctly. Similarly, it should be possible to locate sections (either histological or tomological) of known orientation and hemiretinal origin in standard space.

## Supporting Information

Dataset S1 Complete source code for the Retistruct package (version 0.5.7). Includes demonstration data for Figures 1, 2 and 6 in this paper, a user guide with installation instructions, and a reference manual containing descriptions of the functions used in the package.
(ZIP)

Text S1 Supplemental information including an extended Discussion, and Materials and Methods containing details of experiments and detailed description of the reconstruction algorithm and analysis of reconstructed data.
(PDF)
