# Supplementary material for: Standard Anatomical and Visual Space for the Mouse Retina: Computational Reconstruction and Transformation of Flattened Retinae with the Retistruct Package
Source: PLoS Comput Biol. 2013 Feb 28;9(2):e1002921. doi: 10.1371/journal.pcbi.1002921 (PMC3585388; doi:10.1371/journal.pcbi.1002921)
Supplement: Dataset S1 — Complete source code for the Retistruct package (version 0.5.7). Includes demonstration data for Figures 1, 2 and 6 in this paper, a user guide with installation instructions, and a reference manual containing descriptions of the functions used in the package. (ZIP) [file pcbi.1002921.s001.zip › doc/retistruct-user-guide.pdf]

Retistruct manual

David C. Sterratt

16th November 2012

1 Installation

1.1 Install the necessary system packages

1.1.1 Fedora Linux

As root,

```
yum install R-core
yum install R-devel
yum install gtk2
yum install gtk2-devel
yum install mesa-libGL-devel
yum install mesa-libGLU-devel
```

1.1.2 Ubuntu Linux

At the command line, enter:

```
sudo apt-get install r-base-dev r-base-core r-cran-rgtk2 r-cran-cairodevice r-cran-rgl
```

1.1.3 Mac OS X

First install R for Mac OS X, available from `http://www.r-project.org/`. Under Mac OS X Leopard and Lion, this should be sufficient. Under Mac OS X 10.8 (Snow Leopard), it may be necessary to install the following external packages:

- Xcode (free from App Store)
- Xquartz (http://xquartz.macosforge.org/)
- GTK (http://r.research.att.com/libs/GTK_2.18.5-X11.pkg)

1.1.4 MS/Windows

First install R for MS/Windows, available from `http://www.r-project.org/`.

1.2 Install the core Retistruct package

1. Start R
2. Type:

   ```
   source("http://retistruct.r-forge.r-project.org/install.R")
   ```

   The first time this runs, it should create a personal directory for R packages, and it will take a few minutes to install some required packages.
3. Test that it is working as follows (from within R)

   ```
   library(retistruct)
   demo("retistruct.method")
   ```

If all is well, you should be prompted to press **Return** a few times and Figure 1 from the paper [1] will appear (drawing the 3D renditions takes some time). There will be reports of warnings; they are to do with printing the figures to files. You can view them by typing `warnings()`.

4. A further test produces Figure 2:

   ```
   demo("low.high")
   ```

5. A further (quite long-running) test produces Figure 6:

   ```
   demo("figure6")
   ```

1.3 Install the Retistruct GUI

If the previous step works:

1. Type:

   ```
   source("http://retistruct.r-forge.r-project.org/install-gui.R")
   ```

2. Under MS/Windows, there may be an error message saying that libatk-1.0-0.dll is missing from your computer. Click on **OK**. Another message will appear, asking if you want to install GTK+. Agree to this. After this has finished, quit R (without saving the workspace image) and restart.

3. If all works, an interface window should appear. A number of sets of demonstration data are available from the **Demo** menu item.

2 Running Retistruct

To start the program, start R. At the R prompt type:

```
> library(retistructgui)
> retistruct()
```

A window should appear.

2.1 Opening the files for a retina

There are a number of types of information associated with a flat-mount retina that Retistruct can process:

- The coordinates of the outline of the flattened retina
- An image of the flat-mount retina (optional)
- The coordinates of labelled data points within the flat-mount retina (optional)

To import this information into Retistruct, for each retina, a directory should be created containing files with the above information, as will be described below. At present there are three formats of directory that Retistruct can read. Most users will probably find the the ImageJ ROI format most convenient.

2.1.1 ImageJ ROI format

This format allows you to load images of retinae whose outlines have been marked up in ImageJ. To create the image and outline files for this format:

1. Create a directory to save the files created to.
2. Open up ImageJ (or FIJI).
3. Use **File→Open** to open the image.

4. Use **Image→Scale** to down sample so that the resolution is less than 1000x1000. (This is not crucial, but will speed up things later.)

5. Save the down-sampled version of the image to the file name `image.png` in the directory created in Step 1.

6. Use the Polygon Tool (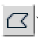) to mark the edge of the retina. According to the IMAGEJ manual[1]: "To create the selection, click repeatedly with the mouse to create line segments. When finished, click in the small box at the starting point (or double-click), and ImageJ automatically draws the last segment.

   The points that define a polygon selection can be moved or deleted, and new points can be added. To delete a point, click on it with the alt key down. To add a point, click on an existing point with the shift key down."

7. Open the ROI manager by selecting **Analyze→Tools→ROI Manager**.

8. Click on the **Add [t]** button.

9. Click on the **Save** button. In the **Save selection...** box that appears, enter the file name `outline.roi` and make sure this file is saved to the same directory as the `image.png` file, i.e. the directory created in Step 1.

10. Open RETISTRUCT.

11. Click on the **Open File** icon and select the directory containing `image.png` and `outline.roi`. The retinal image should now appear, with the outline shown. By default the outline is in black. If this isn't visible against the image, press the **Properties** button in the interface, and change the **Outline colour**.

Specifying the scale Optionally, the scale of the image can be specified by providing a file `scale.csv` in the same directory as the image and ROI files. This file should contain two lines, the first with the heading `Scale` and the second with the length of the side of a pixel in micrometres. For example:

```
"Scale"
1.5
```

Reading in data points In general, the coordinates of data points are read in from a csv file called `datapoints.csv`. The two cells of the first line of the file contain the name of the group of data points and the colour that these should be displayed in RETISTRUCT. Marking up the points from the image in IMAGEJ will ensure that the coordinates are in the same system as those used for the image and the outline. To do this:

1. Open the image used for marking up the outline (`image.png`) in IMAGEJ.

2. Threshold the image (**Image→Adjust→Threshold...** so that the labelled points are visible.

3. Select **Analyze→Set Measurements...** and make sure that **Centroid** is checked

4. Select **Analyze→Analyze Particles**. Make sure that **Display results** and **Clear results** are checked. Click on **OK**.

5. A window entitled **Results** should appear. This contains the X and Y coordinates of the detected points. In this window select **File→Save as...** and save the file as `datapoints.csv`.

6. Open this file with either EXCEL or OPENOFFICE CALC and remove all the columns apart from the X and Y columns. Replace the X with the name of the data set and the Y with the colour it should be displayed in RETISTRUCT.

7. Save `datapoints.csv` in the retina's directory, along with `outline.roi` (and `image.png` if an image is desired).

[1] http://rsbweb.nih.gov/ij/docs/tools.html

2.1.2 CSV format

This is the same as the ImageJ ROI format, except that the outline is contained in the two columns of a file called `outline.csv`. Each column should have a heading, e.g.:

```
"X", "Y"
1,5
10,76,
...
```

2.1.3 IDT format

This is the format used in Ian Thompson's lab (see Appendix A). These files are contained in a directory. To open the files corresponding to a retina, click on the open file icon, and navigate to the directory containing the `SYS` and `MAP` files. On opening this directory, the retinal outline should appear in the Retistruct window.

2.2 The retinal display

In the "Show" section at the bottom left of the screen there are checkboxes that allow you to show various types of information:

Markup Locations of tears and the dorsal or nasal pole

Stitch Locations of how the algorithm has stitched tears (only visible after the reconstruction step)

Grid Lines of latitude and longitude projected back onto the flattened retina (only visible after the reconstruction step)

Datapoints Locations of data points, such as the locations of beads of dye

Landmarks Landmarks such as the optic disc

Strain This shows information about how the retina has been reconstructed, after the reconstruction step has taken place.

Preserve area When this is selected, the polar plot displays an area-preserving projection

Contours Display Kernel Density Estimate contours of the data points. By default, contours at 5%, 25%, 50%, 75% and 95% of the maximum density are shown. This can be changed by a command like this at the R prompt:

```
options(contour.levels=c(5, 25, 50))
```

This command would cause the 5%, 25% and 50% contour lines to be displayed.

2.3 Marking up the retina

Add tear To add a tear, click on this button, then click on three points in turn which define a tear. The order in which the points are added does not matter. Tears contained within a tear can be marked up, but tears cannot cross over one another.

Move Point To move one of the points defining a tear, click on this button, then click on the point which you desire to move, then click on the point to which it should be moved.

Remove tear To remove a tear, click on this button, then click on the apex of the tear (marked in cyan on the plot)

Mark nasal To mark the nasal pole, click on this button, then click on the point which is the nasal pole. If the nasal or dorsal pole has already been marked, the marker is removed from the existing location. The nasal pole should not be in a tear. If the nasal tear is placed within a tear, no error is reported at this stage, but it will be reported later.

Mark dorsal As above, except for the dorsal pole.

Mark OD To mark the optic disc, click on the structure marked in orange which you think is the OD. Once clicked on, the structure should become blue.

Phi0 This determines the latitude of the rim of the reconstructed hemisphere. It depends on the age of the animal.

2.4 Metadata

Data/Flip DV Flip the DV axis to compensate for microscope orientation. Affects display of retinae.

Eye Specify whether the eye is Right or Left. Affects display of retinae.

2.5 Saving the markup and metadata

To save the markup, click on the "Save" button in the toolbar. This saves various markup files to the directory containing the data files. This saved data can be used to reconstruct the retina using a batch process (Section 2.8).

2.6 Reconstructing the retina

To reconstruct the retina, click on the "Reconstruct retina" button. This causes a (lengthy) sequence of operations to be performed:

Stitching Links between corresponding points on parts of the retinal outline contained in tears are made.

Triangulation A triangular mesh is placed over the flattened retina

Initial projection to sphere The mesh is projected roughly onto a sphere

Optimisation The locations of the mesh points on the hemisphere is adjusted so as to minimise a weighted sum of the squared differences between the lengths of links in the mesh on the hemisphere and on the flattened retina, whilst ensuring that as few triangles as possible are flipped.

At the end of the reconstruction process, a polar plot appears next to the flattened retina. When "Landmarks" are shown, the location of the cuts and tears in the polar coordinates can be seen.

When "Strain" is shown, the polar plot is replaced by a scatter plot of the length of links in the reconstructed object versus the length on the flattened object. The colours of the points indicate the degree of expansion or compression from the flattened object to the reconstructed object.

2.7 Saving the reconstruction

To save the markup, click on the "Save" button in the toolbar. This saves various markup files to the directory containing the data files.

2.8 Running a batch of reconstructions

The Retistruct library can be used to reconstruct a batch of retinae which have been marked up. Suppose that the directory `retinae` contains a directory tree in which there are data directories containing `SYS` and `MAP` files and the saved markup files. In order to perform the reconstructions, we create a new directory `retinae/reconstructions`, and run the following sequence of commands in R:

```
R
> library(retistruct)
> retistruct.batch(tldir='retinae', outputdir='retinae/reconstructions')
```

This command will go through the `retinae` directory, looking for valid data directories. If it finds one, it sets about trying to reconstruct the retina. As it reconstructing each retina, it writes to log file in `retinae/reconstructions`. Once the reconstruction is complete, it saves a number of plots in this directory in PDF format. It also adds a line to a summary log file in `retinae/reconstructions` called `retistruct-batch.csv`. This file contains a number of columns:

`Dataset` The directory of the data set

`Return` The return value from the process

`Result` A summary of the result, including if any errors were returned

`E` The total error of the optimised reconstruction

`El` The error due to purely to the lengths of links in the optimised reconstruction

`nflip` The number of flipped triangles

`EOD` The distance of the Optic Disc from the inferred centre of the retina, in degrees. If the OD has not been marked up, this is `NA`.

2.9 Exporting reconstruction data to Matlab

To export the reconstruction data in a directory hierarchy in which `retistruct.batch()` has been run, run the following sequence of commands in R:

```
R
> library(retistruct)
> retistruct.batch.export.matlab(tldir='retinae')
```

This creates a file called `r.mat` in each directory in which there has been a successful reconstruction. To import this data into MATLAB, cd into that directory, and type:

```
clear
load r.mat
```

This puts a number of variables into the workspace, as shown in Table 1.

To produce a polar plot of data points, try the following code:

```
polar(Dss.green(:,2), Dss.green(:,1)*180/pi+90, '.g')
hold on
polar(Dss.red(:,2),   Dss.red(:,1)*180/pi  +90, '.r')
hold off
```

The radial axis indicates the latitude in degrees measured from the retinal pole.

In the `matlab` subdirectory of the distribution, there are some scripts to produce polar polar plots, including the locations of tears and landmarks. To create PDF plots of all the retinae in a directory, try:

```
makefigures('retinae', 'output_directory')
```

There are also some embryonic scripts to create polar plots: `plot_datapoints_polararea.m` and `polararea.m`.

A Data format

The data for each retina is stored in a separate directory. Within each directory there are two files:

`SYS.SYS` A table in SYSTAT format containing the coordinates of the red, green and doubly labelled cell bodies, and counts of labelled cell bodies within each grid box. The column headings shown in Table 2. Each row of the table contains information only on a subset of the data, e.g. the coordinates of a red-labelled cell.

`ALU.MAP` A text file containing the coordinates of the map outline. The file comprises a number of sections, each starting with a single number, which is the number of lines to read in the next section. These lines have two numbers each, the x and y coordinates of a vertex of the map outline.

References

[1] Sterratt DC, Lyngholm D, Willshaw DJ, Thompson ID (in press) Standard anatomical and visual space for the mouse retina: computational reconstruction and transformation of flattened retinae with the Retistruct package PLoS Comp. Biol.

`phi0`	The latitude of the rim, expressed in radians	
`Dss.green`, `Dss.red`	Locations of green (red) labelled cell bodies in spherical coordinates. The first column contains the latitude of each point, measured in radians. The second column contains the longitude of each point, measured in radians.	
`DssMean.green`, `DssMean.red`	Location of Karcher mean of green-labelled cell bodies in spherical coordinates. The first column contains the latitude of each point, measured in radians. The second column contains the longitude of each point, measured in radians.	
`DssHullarea.green`, `DssHullarea.red`	Area of convex hull of green (red) points on sphere. The convex hull is essentially a polygon drawn around data points.	
`Tss`	A structure in which each element contains spherical coordinates (in the same latitude-longitude format as above) of a tear.	
`Sss`	A structure in which each element contains spherical coordinates (in the same latitude-longitude format as above) of a landmark.	
`KDE`	An object containing information about Kernel Density Estimates of the locations of cell bodies.	
	`green_flevels`	Contour heights, determined by finding heights that exclude a certain fraction of the probability. For example, the 95% contour is excludes 95% of the probability mass, and it should enclose about 5% of the points.
	`green_labels`	Contour labels. These give the label (e.g. 5, 25, 50, where these are the percentages above) of each contour. Note that there may be more than one contour at the same level, so this vector may contain more elements than `flevels`. The first element of `green_labels` labels the contour whose coordinates are specified in `green_contours1`, the second element of `green_labels` relates to `green_contours2` and so on.
	`green_tot_contour_areas`	The total area in square degrees enclosed by each contour. This is a matrix with the first column giving the contour label (see above) and the next column giving the area.
	`green_kappa`	The concentration parameter of the Fisher density determined by the kernel fitting algorithm.
	`green_h`	A pseudo-bandwidth parameter, the inverse of the square root of `kappa`. Units of degrees.
	`green_maxs_phi`	Lattitude of maximum point of kernel estimate.
	`green_maxs_lambda`	Longitude of maximum point of kernel estimate.
	`green_g_xs` `green_g_ys` `green_g_f`	Kernel density estimates on standard polar grid. This can be plotted in MATLAB using the command `contour(KDE.green_g_xs, KDE.green_g_ys, KDE.green_g_f)`.
	`green_gpa_xs` `green_gpa_ys` `green_gpa_f`	Kernel density estimates on area-preserving polar grid. Plotted in MATLAB as above.
	`green_contours1`, `green_contours2`...	Coordinates of contours. See `green_labels` above for more explanation.
	`green_contour_areas1`, `green_contour_areas2`...	Area contained within each individual contour. See `green_labels` above for more explanation.
`KR`	An object containing Kernel Regression estimates of the density of points, derived from the grouped data points. All fields correspond to `KDE` above.	

Table 1: Variables exported in the `r.mat` file.

**FOR EACH BOUNDARY**	
`MAPNUM`	id number of boundary
`MINLAT`	min latitude
`MAXLAT`	max latitude
`MINLON`	min longitude
`MAXLON`	max longitude
`LABLAT`	latitude of label
`LABLON`	longitude of label
**FOR EACH CELL**	
`XRED`	x-coordinate if cell labelled red but not doubly
`YRED`	y-coordinate if cell labelled red but not doubly
`XGREEN`	x-coordinate if cell labelled green but not doubly
`YGREEN`	y-coordinate if cell labelled green but not doubly
`XDOUBLE`	x-coordinate if cell labelled doubly
`YDOUBLE`	y-coordinate if cell labelled doubly
`XGRID`	sample box cell is in
`YGRID`	sample box cell is in
`PERIM`	perimeter of cell
`AREA`	area of cell
**ONE PER GRID BOX**	
`GRIDX`	grid location of centre of sample box
`GRIDY`	grid location of centre of sample box
`XGRIDCOO`	x-coordinate of centre of sample box
`YGRIDCOO`	y-coordinate of centre of sample box
`BOXSIZEX`	size (half width) of sample box in x-direction
`BOXSIZEY`	size (half width) of sample box in y-direction
`COMPLETE`	whether counting of sample has been completed
`TOTALCEL`	total number of cells in this box
`TOTALRED`	total number of red-only cells in this box
`TOTALGRE`	total number of green-only cells in this box
`TOTALDOU`	total number of double cells in this box
`MEANPERI`	average perimeter of all cells
`MEANAREA`	average area of all cells

Table 2: Column headings of the `SYS.SYS` file.
