# Supplementary material for: Standard Anatomical and Visual Space for the Mouse Retina: Computational Reconstruction and Transformation of Flattened Retinae with the Retistruct Package
Source: PLoS Comput Biol. 2013 Feb 28;9(2):e1002921. doi: 10.1371/journal.pcbi.1002921 (PMC3585388; doi:10.1371/journal.pcbi.1002921)
Supplement: Dataset S1 — Complete source code for the Retistruct package (version 0.5.7). Includes demonstration data for Figures 1, 2 and 6 in this paper, a user guide with installation instructions, and a reference manual containing descriptions of the functions used in the package. (ZIP) [file pcbi.1002921.s001.zip › doc/retistruct-manual.pdf]

Package ‘retistruct’

November 16, 2012

Maintainer David C. Sterratt <david.c.sterratt@ed.ac.uk>

License GPL-2

Title Retinal reconstruction program

Author David C. Sterratt <david.c.sterratt@ed.ac.uk>

Description Program to reconstruct retinae from flattened retinae in which there are incisions and tears

Version 0.5.7

URL http://www.neuralmapformation.org

Date 2012-11-16

Depends
R (>= 2.14.0), geometry (>= 0.2-1), foreign, R.matlab,ttutils, RTriangle (>= 1.6-0.4), rgl, png, sp

Suggests multicore

Collate 'AnnotatedOutline.R' 'Dataset.R' 'fire.R' 'format-common.R' 'format-csv.R' 'format-idt.R' 'format-ijroi.R' 'generics.R' 'geometry.R' 'kernel-density.R' 'misc.R' 'Outline.R' 'plots.R' 'ReconstructedDataset.R' 'ReconstructedOutline.R' 'RetinalDataset.R' 'RetinalReconstructedDataset.R' 'RetinalReconstructedOutline.R' 'retistruct-batch.R' 'retistruct-cli.R' 'retistruct.R' 'revision.R' 'roiread.R' 'spheristruct.R' 'StitchedOutline.R' 'TriangulatedOutline.R' 'zzz.R' 'ray-tracing.R' 'projections.R' 'transformations.R'

R topics documented:

addClass 4
addTear 5
AnnotatedOutline 6
azel.to.sphere.colattitude 6
azimuthal.conformal 7
azimuthal.equalarea 8
azimuthal.equidistant 8
bary.to.sphere.cart 9
central.angle 10
checkDatadir 10

checkTears . . . 11
circle . . . 11
compute.intersections.sphere . . . 12
compute.kernel.estimate . . . 13
computeTearRelationships . . . 14
create.polar.cart.grid . . . 14
csv.read.dataset . . . 15
Dataset . . . 16
dE . . . 16
draw.arc . . . 17
draw.system . . . 18
E . . . 18
Ecart . . . 19
ensureFixedPointInRim . . . 20
f . . . 21
Fcart . . . 21
fire . . . 22
flatplot . . . 23
flatplot.annotatedOutline . . . 24
flatplot.dataset . . . 24
flatplot.outline . . . 25
flatplot.reconstructedOutline . . . 25
flatplot.retinalDataset . . . 26
flatplot.stitchedOutline . . . 27
flatplot.triangulatedOutline . . . 27
flipped.triangles . . . 28
flipped.triangles.cart . . . 28
fp . . . 29
getDss . . . 30
getDss.reconstructedDataset . . . 30
getDss.retinalReconstructedDataset . . . 31
getDssHullarea . . . 31
getDssMean . . . 32
getDssMean.reconstructedDataset . . . 32
getDssMean.retinalReconstructedDataset . . . 33
getGss . . . 33
getGss.reconstructedDataset . . . 34
getGss.retinalReconstructedDataset . . . 34
getIDs . . . 35
getIDs.dataset . . . 35
getIDs.reconstructedDataset . . . 36
getIms . . . 36
getIms.reconstructedOutline . . . 37
getKDE . . . 37
getKR . . . 38
getSss . . . 38
getSss.reconstructedDataset . . . 39
getSss.retinalReconstructedDataset . . . 39
getStrains . . . 40
getTear . . . 40
getTss.reconstructedOutline . . . 41
getTss.retinalReconstructedOutline . . . 41

identity.transform . . . 42
idt.read.dataset . . . 42
ijroi.read.dataset . . . 43
incident.to.lens.angle . . . 44
invert.sphere . . . 45
invert.sphere.to.hemisphere . . . 45
karcher.mean.sphere . . . 46
kde.compute.concentration . . . 47
kde.fhat . . . 47
kde.fhat.cart . . . 48
kde.L . . . 48
kr.compute.concentration . . . 49
kr.sscv . . . 49
kr.yhat . . . 50
kr.yhat.cart . . . 50
labelTearPoints . . . 51
line.line.intersection . . . 51
list.datasets . . . 52
lvsLplot.reconstructedOutline . . . 53
mergePointsEdges . . . 53
name.list . . . 54
nameLandmark.dataset . . . 55
nameLandmark.retinalDataset . . . 55
new.ray . . . 56
normalise.angle . . . 56
optimiseMapping . . . 57
orthographic . . . 58
Outline . . . 58
panlabel . . . 59
plot.IJROI . . . 59
polar.cart.to.sphere.spherical . . . 60
polartext . . . 61
projection . . . 61
projection.reconstructedDataset . . . 62
projection.reconstructedOutline . . . 63
projectToSphere . . . 64
ray.arc.intersection . . . 64
Rcart . . . 65
read.datapoints . . . 66
read.ijroi . . . 66
ReconstructedDataset . . . 67
ReconstructedOutline . . . 68
remove.identical.consecutive.rows . . . 69
remove.intersections . . . 70
removeTear . . . 70
RetinalDataset . . . 71
RetinalReconstructedDataset . . . 71
RetinalReconstructedOutline . . . 72
retistruct.batch . . . 72
retistruct.batch.analyse.summaries . . . 73
retistruct.batch.analyse.summary . . . 74
retistruct.batch.export.matlab . . . 74

retistruct.batch.figures . . . 75
retistruct.batch.plot.ods . . . 75
retistruct.batch.summary . . . 76
retistruct.check.markup . . . 76
retistruct.cli . . . 77
retistruct.cli.figure . . . 77
retistruct.cli.process . . . 78
retistruct.export.matlab . . . 79
retistruct.potential.od . . . 79
retistruct.read.dataset . . . 80
retistruct.read.markup . . . 80
retistruct.read.recdata . . . 81
retistruct.reconstruct . . . 82
retistruct.save.markup . . . 82
retistruct.save.recdata . . . 83
rotate.axis . . . 83
setFixedPoint . . . 84
simplify.outline . . . 85
sinusoidal . . . 85
solveMappingCart . . . 86
sphere.cart.to.sphere.spherical . . . 87
sphere.spherical.to.polar.cart . . . 88
sphere.spherical.to.sphere.cart . . . 88
sphere.tri.area . . . 89
spherical.to.polar.area . . . 90
sphericalplot.reconstructedDataset . . . 91
sphericalplot.reconstructedOutline . . . 91
StitchedOutline . . . 92
strain.colours . . . 93
stretchMesh . . . 93
titrate.reconstructedOutline . . . 94
trace.ray . . . 94
trace.ray.angle . . . 95
transform.image.reconstructedOutline . . . 95
tri.area . . . 96
tri.area.signed . . . 97
TriangulatedOutline . . . 97
vecnorm . . . 98
whichTear . . . 99

Index **100**

`addClass` *Add new class to class vector of object*

Description

This adds a new class to the class vector. If the class is dataset type it is prepended at the start of the class vector. If it is an outline type, it is put after all the dataset classes, but before the other outline ones. This is needed for the plotting functions to work properly.

Usage

```
addClass(newclass, obj)
```

Arguments

`newclass`	New class to add
`obj`	Object to which to add it

Value

New class vector

Author(s)

David Sterratt

`addTear` *Add tear to an AnnotatedOutline*

Description

Add tear to an AnnotatedOutline

Usage

```
addTear(a, pids)
```

Arguments

`a`	`AnnotatedOutline` object
`pids`	Vector of three point IDs to be added

Value

`AnnotatedOutline` object

Author(s)

David Sterratt

AnnotatedOutline *Constructor for AnnotatedOutline object*

Description

Constructor for AnnotatedOutline object.

Usage

```
AnnotatedOutline(o)
```

Arguments

o	`Outline` object

Value

AnnotatedOutline object, with extra fields for tears (`V0`, `VF` and `VB`), lattitude of rim `phi0` and index of fixed point `i0`.

Author(s)

David Sterratt

azel.to.sphere.colattitude *Convert azimuth-elevation coordinates to spherical coordinates*

Description

Convert azimuth-elevation coordinates to spherical coordinates

Usage

```
azel.to.sphere.colattitude(r, r0)
```

Arguments

r	Coordinates of points in azimuth-elevation coordinates represented as 2 column matrix with column names `alpha` (elevation) and `theta` (azimuth).
r0	Direction of the axis of the sphere on which to project represented as a 2 column matrix of with column names `alpha` (elevation) and `theta` (azimuth).

Value

2-column matrix of spherical coordinates of points with column names `psi` (colattidude) and `lambda` (longitude).

Author(s)

David Sterratt

Examples

```
r0 <- cbind(alpha=0, theta=0)
r <- rbind(r0, r0+c(1,0), r0-c(1,0), r0+c(0,1), r0-c(0,1))
azel.to.sphere.colattitude(r, r0)
```

`azimuthal.conformal` *Azimuthal conformal or stereographic or Wulff projection*

Description

Azimuthal conformal or stereographic or Wulff projection

Usage

```
azimuthal.conformal(r, ...)
```

Arguments

`r`	2-column Matrix of spherical coordinates of points on sphere. Column names are `phi` and `lambda`.
`...`	Arguments not used by this projection.

Value

2-column Matrix of Cartesian coordinates of points on polar projection. Column names should be `x` and `y`.

Note

This is a special case with the point centred on the projection being the South Pole. The Mathworld equations are for the more general case.

Author(s)

David Sterratt

References

http://en.wikipedia.org/wiki/Map_projection, http://mathworld.wolfram.com/StereographicProjection.html Fisher, N. I., Lewis, T., and Embleton, B. J. J. (1987). Statistical analysis of spherical data. Cambridge University Press, Cambridge, UK.

`azimuthal.equalarea` *Lambert azimuthal equal area projection*

Description

Lambert azimuthal equal area projection

Usage

```
azimuthal.equalarea(r, ...)
```

Arguments

`r`	2-column Matrix of spherical coordinates of points on sphere. Column names are `phi` and `lambda`.
`...`	Arguments not used by this projection.

Value

2-column Matrix of Cartesian coordinates of points on polar projection. Column names should be `x` and `y`.

Note

This is a special case with the point centred on the projection being the South Pole. The Mathworld equations are for the more general case.

Author(s)

David Sterratt

References

http://en.wikipedia.org/wiki/Map_projection, http://mathworld.wolfram.com/LambertAzimuthalEqual-AreaProjection.html Fisher, N. I., Lewis, T., and Embleton, B. J. J. (1987). Statistical analysis of spherical data. Cambridge University Press, Cambridge, UK.

`azimuthal.equidistant` *Azimuthal equidistant projection*

Description

Azimuthal equidistant projection

Usage

```
azimuthal.equidistant(r, ...)
```

Arguments

`r`	2-column Matrix of spherical coordinates of points on sphere. Column names are `phi` and `lambda`.
`...`	Arguments not used by this projection.

Value

2-column Matrix of Cartesian coordinates of points on polar projection. Column names should be `x` and `y`.

Note

This is a special case with the point centred on the projection being the South Pole. The Mathworld equations are for the more general case.

Author(s)

David Sterratt

References

http://en.wikipedia.org/wiki/Map_projection, http://mathworld.wolfram.com/AzimuthalEquidistantProjection.html

---

`bary.to.sphere.cart` *Convert barycentric coordinates of points in mesh on sphere to cartesian coordinates*

Description

Given a triangular mesh on a sphere described by mesh locations (`phi`, `lambda`), a radius `R` and a triangulation `Tt`, determine the Cartesian coordinates of points `cb` given in barycentric coordinates with respect to the mesh.

Usage

```
bary.to.sphere.cart(phi, lambda, R, Tt, cb)
```

Arguments

`phi`	Lattitudes of mesh points
`lambda`	Longitudes of mesh points
`R`	Radius of sphere
`Tt`	Triagulation
`cb`	Object returned by tsearch containing information on the triangle in which a point occurs and the barycentric coordinates within that triangle

Value

An N-by-3 matrix of the Cartesian coordinates of the points

Author(s)

David Sterratt

`central.angle` *Central angle between two points on a sphere*

Description

On a sphere the central angle between two points is defined as the angle whose vertex is the centre of the sphere and that subtends the arc formed by the great circle between the points. This function computes the central angle for two points (ϕ_1, λ_1) and (ϕ_2, λ_2).

Usage

```
central.angle(phi1, lambda1, phi2, lambda2)
```

Arguments

`phi1`	Lattitude of first point
`lambda1`	Longitude of first point
`phi2`	Lattitude of second point
`lambda2`	Longitude of secone point

Value

Central angle

Author(s)

David Sterratt

Source

Wikipedia `http://en.wikipedia.org/wiki/Central_angle`

`checkDatadir` *Check the whether directory contains valid data*

Description

Check the whether directory contains valid data

Usage

```
checkDatadir(dir = NULL)
```

Arguments

`dir`	Diectory to check.

Value

`TRUE` if `dir` contains valid data; `FALSE` otherwise.

Author(s)

David Sterratt

`checkTears` *Check that tears are all in the correct direction*

Description

Given a tear matrix T with columns "V0", "VF", and "VB", check that all tears are correct.

Usage

```
checkTears(o)
```

Arguments

`o`	`AnnotatedOutline` object

Value

If all is OK, returns empty vector. If not, returns indicies of problematic tears.

Author(s)

David Sterratt

`circle` *Return points on the unit circle*

Description

Return points on the unit circle in an anti-clockwise direction. If `L` is not specified `n` points are returned. If `L` is specified, the same number of points are returned as there are elements in `L`, the interval between successive points being proportional to `L`.

Usage

```
circle(n = 12, L = NULL)
```

Arguments

`n`	Number of points
`L`	Intervals between points

Value

The cartesian coordinates of the points

Author(s)

David Sterratt

`compute.intersections.sphere`
Find the intersection of a plane with triangles on a sphere

Description

Find the interections of the plane defined by the normal `n` and the distance `d` expressed as a fractional distance along the side of each triangle.

Usage

```
compute.intersections.sphere(phi, lambda, T, n, d)
```

Arguments

`phi`	Lattitude of grid points on sphere centred on origin.
`lambda`	Longitude of grid points on sphere centred on origin.
`T`	Triangulation
`n`	Normal of plane
`d`	Distance of plane along normal from origin.

Value

Matrix with same dimensions as `T`. Each row gives the intersection of the plane with the corresponding triangle in `T`. Column 1 gives the fractional distance from vertex 2 to vertex 3. Column 2 gives the fractional distance from vertex 3 to vertex 1. Column 2 gives the fractional distance from vertex 1 to vertex 2. A value of `NaN` indicates that the corresponding side lies in the plane. A value of `Inf` indicates that the side lies parallel to the plane but outside it.

Author(s)

David Sterratt

`compute.kernel.estimate` *Kernel estimate over grid*

Description

Compute a kernel estimate over a grid and do a contour analsysis of this estimate. The contour heights the determined by finding heights that exclude a certain fraction of the probability. For example, the 95 is excludes 95 enclose about 5 specified by the `contour.levels` option; by default they are `c(5, 25, 50, 75, 95)`.

Usage

```
compute.kernel.estimate(Dss, phi0, fhat, compute.conc)
```

Arguments

`Dss`	List of datasets. The first two columns of each datasets are coordinates of points on the sphere in spherical polar (lattitude, `phi`, and longitude, `lambda`) coordinates. In the case kernel smoothing, there is a third column of values of dependent variables at those points.
`phi0`	Rim angle in radians
`fhat`	Function such as `kde.fhat` or `kr.yhat` to compute the density given data and a value of the concentration parameter `kappa` of the Fisher density.
`compute.conc`	Function to return the optimal value of the concentration parameter kappa given the data.

Value

A list containing

`kappa`	The concentration parameter
`h`	A pseudo-bandwidth parameter, the inverse of the square root of `kappa`. Units of degrees.
`flevels`	Contour levels.
`labels`	Labels of the contours.
`g`	Raw density estimate drawn on non-area-preserving projection. Comprises locations of gridlines in Cartesian coordinates (`xs` and `ys`), density estimates at these points, `f` and location of maximum in Cartesian coordinates (`max`).
`gpa`	Raw density estimate drawn on area-preserving projection. Comprises same elements as above.
`contour.areas`	Area of each individual contour. One level may ahave more than one contour; this shows the areas of all such contours.
`tot.contour.areas`	Data frame containing the total area within the contours at each level.

Author(s)

David Sterratt

`computeTearRelationships` *Compute the parent relationships for a set of tears*

Description

Compute the parent relationships for a potential set of tears on an `AnnotatedOutline`. The function throws an error if tears overlap.

Usage

```
computeTearRelationships(o, V0, VB, VF)
```

Arguments

`o`	`AnnotatedOutline` object
`V0`	Apices of tears
`VB`	Backward vertices of tears
`VF`	Forward vertices of tears

Value

List

`Rset`	the set of points on the rim
`TFset`	list containing indicies of points in each foward tear
`TBset`	list containing indicies of points in each backward tear
`h`	correspondence mapping
`hf`	correspondence mapping in forward direction for points on boundary
`hb`	correspondence mapping in backward direction for points on boundary

Author(s)

David Sterratt

`create.polar.cart.grid` *Create grid on projection of hemisphere onto plane*

Description

Create grid on projection of hemisphere onto plane

Usage

```
create.polar.cart.grid(pa, res, phi0)
```

Arguments

pa	If TRUE, make this an area-preserving projection
res	Resolution of grid
phi0	Value of phi0 at edge of grid

Value

List containing:

s	Grid locations in spherical coordinates
c	Grid locations in Cartesian coordinates on plane
xs	X grid line locations in Cartesian coordinates on plane
ys	Y grid line locations in Cartesian coordinates on plane

Author(s)

David Sterratt

---

csv.read.dataset *Read a retinal dataset in CSV format*

Description

Read a retinal dataset in CSV format. Each dataset is a folder containing a file called outline.csv that specifies the outline in X-Y coordinates. It may also contain a file datapoints.csv, containing the locations of data points; see read.datapoints for the format of this file.

Usage

```
csv.read.dataset(dataset)
```

Arguments

dataset	Path to directory containing outline.csv

Value

A RetinalDataset object

Author(s)

David Sterratt

`Dataset` *Constructor for a* `dataset` *object.*

Description

Constructor for a `dataset` object.

Usage

```
Dataset(o, dataset, Ds, Ss, cols, raw, Gs = NULL)
```

Arguments

`o`	An `outline` object.
`dataset`	The name of the dataset
`Ds`	A list of data point sets, with each set being a 2 column matrix of X and Y coordinates of data point locations. Each item in the list should be named. Elements with these names should also be in the `cols` argument (see below).
`Ss`	A list of landmarks. These do not need to be named. If any elements are named, the names should map onto an element in the `cols` argument. Any elements that are named `""` will be plotted using the default colour.
`cols`	A list of colours in which to plot datapoints and landmarks.
`raw`	A place to put raw data in whatever format is desired.
`Gs`	A list of grouped point sets, with each set being a 3 column matrix of X and Y coordinates and the value Z of the variable at that point. Each item in the list should be named. Elements with these names should also be in the `cols` argument.

Value

A `dataset` object.

Author(s)

David Sterratt

`dE` *The deformation energy gradient function*

Description

The function that computes the gradient of the energy (or error) of the deformation of the mesh from the flat outline to the sphere. This depends on the locations of the points given in spherical coordinates. The function is designed to take these as a vector that is received from the `optim` function.

Usage

```
dE(p, Cu, C, L, B, T, A, R, Rset, i0, phi0, lambda0,
  Nphi, N, alpha = 1, x0, nu = 1, verbose = FALSE)
```

Arguments

`p`	Parameter vector of `phi` and `lambda`
`Cu`	The upper part of the connectivity matrix
`C`	The connectivity matrix
`L`	Length of each edge in the flattened outline
`B`	Connectivity matrix
`T`	Triangulation in the flattened outline
`A`	Area of each triangle in the flattened outline
`R`	Radius of the sphere
`Rset`	Indicies of points on the rim
`i0`	Index of fixed point on rim
`phi0`	Lattitude at which sphere curtailed
`lambda0`	Longitude of fixed points
`Nphi`	Number of free values of `phi`
`N`	Number of points in sphere
`alpha`	Area penalty scaling coefficient
`x0`	Area penalty cutoff coefficient
`nu`	Power to which to raise area
`verbose`	How much information to report

Value

A vector representing the derivative of the energy of this particular configuration with respect to the parameter vector

Author(s)

David Sterratt

`draw.arc` *Draw a arc*

Description

Draw a arc

Usage

```
draw.arc(A, R, C = pi/2, ...)
```

Arguments

`A`	Left edge of arc
`R`	Radius of arc
`C`	Cutoff angle
`...`	Graphics parameters

Author(s)

David Sterratt

`draw.system` *Draw a system of lenses*

Description

Draw a system of lenses

Usage

```
draw.system(S, xoff = -10, ypad = 1, add = FALSE, ...)
```

Arguments

`S`	System defined by data frame containing left margin of surfaces (A), radii of surfaces (R) and refracive indices (n).
`xoff`	x offset of left edge
`ypad`	y padding
`add`	If `TRUE` add to an existing plot
`...`	Graphics parameters

Author(s)

David Sterratt

`E` *The deformation energy function*

Description

The function that computes the energy (or error) of the deformation of the mesh from the flat outline to the sphere. This depends on the locations of the points given in spherical coordinates. The function is designed to take these as a vector that is received from the `optim` function.

Usage

```
E(p, Cu, C, L, B, T, A, R, Rset, i0, phi0, lambda0, Nphi,
  N, alpha = 1, x0, nu = 1, verbose = FALSE)
```

Arguments

`p`	Parameter vector of `phi` and `lambda`
`Cu`	The upper part of the connectivity matrix
`C`	The connectivity matrix
`L`	Length of each edge in the flattened outline
`B`	Connectivity matrix
`T`	Triangulation in the flattened outline
`A`	Area of each triangle in the flattened outline
`R`	Radius of the sphere
`Rset`	Indicies of points on the rim
`i0`	Index of fixed point on rim
`phi0`	Lattitude at which sphere curtailed
`lambda0`	Longitude of fixed points
`Nphi`	Number of free values of `phi`
`N`	Number of points in sphere
`alpha`	Area scaling coefficient
`x0`	Area cutoff coefficient
`nu`	Power to which to raise area
`verbose`	How much information to report

Value

A single value, representing the energy of this particular configuration

Author(s)

David Sterratt

`Ecart` *The deformation energy function*

Description

The function that computes the energy (or error) of the deformation of the mesh from the flat outline to the sphere. This depends on the locations of the points given in spherical coordinates. The function is designed to take these as a vector that is received from the `optim` function.

Usage

```
Ecart(P, Cu, L, T, A, R, alpha = 1, x0, nu = 1,
  verbose = FALSE)
```

Arguments

`P`	N-by-3 matrix of point coordinates
`Cu`	The upper part of the connectivity matrix
`L`	Length of each edge in the flattened outline
`T`	Triangulation in the flattened outline
`A`	Area of each triangle in the flattened outline
`R`	Radius of sphere
`alpha`	Area penalty scaling coefficient
`x0`	Area penalty cutoff coefficient
`nu`	Power to which to raise area
`verbose`	How much information to report

Value

A single value, representing the energy of this particular configuration

Author(s)

David Sterratt

`ensureFixedPointInRim` *Ensure that the fixed point is in the rim, not a tear*

Description

Ensure that the fixed point `i0` is in the rim, not a tear.

Usage

```
ensureFixedPointInRim(o)
```

Arguments

`o`	`AnnotatedOutline` object

Value

o `AnnotatedOutline` object in which `i0` may have been changed.

Author(s)

David Sterratt

f *Piecewise smooth function used in area penalty*

Description

Piecewise, smooth function that increases linearly with negative arguments.

$$f(x) = \begin{cases} -(x - x_0/2) & x < 0 \\ \frac{1}{2x_0}(x - x_0)^2 & 0 < x < x_0 \\ 0 & x \geq x_0 \end{cases}$$

Usage

```
f(x, x0)
```

Arguments

x	Main argument
x0	The cutoff parameter. Above this value the function is zero.

Value

The value of the function.

Author(s)

David Sterratt

Fcart *The deformation energy gradient function*

Description

The function that computes the gradient of the energy (or error) of the deformation of the mesh from the flat outline to the sphere. This depends on the locations of the points given in spherical coordinates. The function is designed to take these as a vector that is received from the `optim` function.

Usage

```
Fcart(P, C, L, T, A, R, alpha = 1, x0, nu = 1,
  verbose = FALSE)
```

Arguments

`P`	N-by-3 matrix of point coordinates
`C`	The connectivity matrix
`L`	Length of each edge in the flattened outline
`T`	Triangulation in the flattened outline
`A`	Area of each triangle in the flattened outline
`R`	Radius of sphere
`alpha`	Area penalty scaling coefficient
`x0`	Area penalty cutoff coefficient
`nu`	Power to which to raise area
`verbose`	How much information to report

Value

A vector representing the derivative of the energy of this particular configuration with respect to the parameter vector

Author(s)

David Sterratt

`fire` *The FIRE algorithm*

Description

This is an implmentation of the FIRE algorithm for structural relaxation put forward by Bitzek et al. (2006)

Usage

```
fire(r, force, restraint, m = 1, dt = 0.1, maxmove = 100,
  dtmax = 1, Nmin = 5, finc = 1.1, fdec = 0.5,
  astart = 0.1, fa = 0.99, a = 0.1, nstep = 100,
  tol = 1e-05, verbose = FALSE)
```

Arguments

`r`	Initial locations of particles
`force`	Force function
`restraint`	Restraint function
`m`	Masses of points
`dt`	Initial time step
`maxmove`	Maximum distance to move in any time step
`dtmax`	Maxiumum time step
`Nmin`	Number of steps after which to start increasing `dt`

finc	Fractional increase in dt per time step
fdec	Fractional decrease in dt after a stop
astart	Starting value of a after a stop
fa	Fraction of a to retain after each step
a	Initial value of a
nstep	Maxiumum number of steps
tol	Tolerance - if RMS force is below this value, stop and report convergence
verbose	If TRUE report progress verbosely

Value

List containing x, the positions of the points, conv, which is 0 if convergence as occured and 1 otherwise, and frms, the root mean square of the forces on the particles.

Author(s)

David Sterratt

References

Bitzek, E., Koskinen, P., G\"ahler, F., Moseler, M., and Gumbsch, P. (2006). Structural relaxation made simple. Phys. Rev. Lett., 97:170201.

---

flatplot *Flat plot of object*

Description

Plot flat representation of object

Usage

```
flatplot(x, axt = "n", ylim = NULL, ...)
```

Arguments

x	Outline, Dataset \&c object
axt	whether to plot axes
ylim	y-limits
...	Other plotting parameters

Author(s)

David Sterratt

flatplot.annotatedOutline *Flat plot of AnnotatedOutline*

Description

Plot flat AnnotatedOutline. The user markup is displayed by default.

Usage

```
## S3 method for class 'annotatedOutline'
flatplot(x, axt = "n",
  ylim = NULL, markup = TRUE, ...)
```

Arguments

x	AnnotatedOutline object
axt	whether to plot axes
ylim	y-limits
markup	If TRUE, plot markup
...	Other plotting parameters

Author(s)

David Sterratt

flatplot.dataset *Flat plot of Dataset*

Description

Flat plot of Dataset

Usage

```
## S3 method for class 'dataset'
flatplot(x, axt = "n", ylim = NULL,
  datapoints = TRUE, grouped = FALSE, landmarks = TRUE,
  ids = getIDs(x), ...)
```

Arguments

x	Dataset object
axt	whether to plot axes
ylim	y-limits
datapoints	If TRUE, display data points.
grouped	If TRUE, dipslay grouped data.
landmarks	If TRUE, dipslay landmarks.
ids	IDs of groups of data within a dataset, returned using getIDs.
...	Graphical parameters to pass to plotting functions

Author(s)

David Sterratt

`flatplot.outline` *Flat plot of outline*

Description

Plot flat `Outline`.

Usage

```
## S3 method for class 'outline'
flatplot(x, axt = "n", ylim = NULL,
  image = TRUE, scalebar = 1, add = FALSE, ...)
```

Arguments

`x`	`Outline` object
`axt`	whether to plot axes
`ylim`	y-limits
`image`	If `TRUE` the image (if it is present) is displayed behind the outline
`scalebar`	If numeric and if the Outline has a `scale` field, a scale bar of length `scalebar` mm is plotted. If `scalebar` is `FALSE` or there is no scale information in the `Outline` `x` the scale bar is supressed.
`add`	If `TRUE`, don't draw axes; add to existing plot.
`...`	Other plotting parameters

Author(s)

David Sterratt

`flatplot.reconstructedOutline`
Flat plot of reconstructed outline

Description

Plot `ReconstructedOutline` object. This adds a mesh of gridlines from the spherical retina (described by points `phi`, `lambda` and triangulation `Tt` and cutoff point `phi0`) onto a flattened retina (described by points `P` and triangulation `T`).

Usage

```
## S3 method for class 'reconstructedOutline'
flatplot(x, axt = "n",
  ylim = NULL, grid = TRUE, strain = FALSE, ...)
```

Arguments

`x`	`ReconstructedOutline` object
`axt`	whether to plot axes
`ylim`	y-limits
`grid`	Whether or not to show the grid lines of lattitude and longitude
`strain`	Whether or not to show the strain
`...`	Other plotting parameters

Author(s)

David Sterratt

`flatplot.retinalDataset` *Flat plot of retinal dataset*

Description

Plot an retinal dataset. This basically is equivalent to plotting a `dataset`, but may perform some transformations to the date or plotting parameters. At present, if `DVflip` is `TRUE`, it flips the *y*-axis.

Usage

```
## S3 method for class 'retinalDataset'
flatplot(x, axt = "n",
  ylim = NULL, ...)
```

Arguments

`x`	`retinalDataset` object
`axt`	whether to plot axes
`ylim`	y-limits
`...`	Other plotting parameters

Author(s)

David Sterratt

`flatplot.stitchedOutline`
Flat plot of AnnotatedOutline

Description

Plot flat `StitchedOutline`. If the optional argument `stitch` is `TRUE` the user markup is displayed.

Usage

```
## S3 method for class 'stitchedOutline'
flatplot(x, axt = "n",
  ylim = NULL, stitch = TRUE, ...)
```

Arguments

`x`	`AnnotatedOutline` object
`axt`	whether to plot axes
`ylim`	y-limits
`stitch`	If `TRUE`, plot stitch
`...`	Other plotting parameters

Author(s)

David Sterratt

`flatplot.triangulatedOutline`
Flat plot of TriangulatedOutline

Description

Plot flat `TriangulatedOutline`.

Usage

```
## S3 method for class 'triangulatedOutline'
flatplot(x, axt = "n",
  ylim = NULL, mesh = TRUE, ...)
```

Arguments

`x`	`TriangulatedOutline` object
`axt`	whether to plot axes
`ylim`	y-limits
`mesh`	If `TRUE`, plot mesh
`...`	Other plotting parameters

Author(s)

David Sterratt

`flipped.triangles` *Determine indicies of triangles that are flipped*

Description

In the projection of points onto the sphere, some triangles maybe flipped, i.e. in the wrong orientation. This functions determines which triangles are flipped by computing the vector pointing to the centre of each triangle and comparing this direction to vector product of two sides of the triangle.

Usage

```
flipped.triangles(phi, lambda, Tt, R)
```

Arguments

`phi`	Vector of lattitudes of points
`lambda`	Vector of longitudes of points
`Tt`	Triangulation of points
`R`	Radius of sphere

Value

List containing:

`flipped`	Indicies of in rows of `Tt` of flipped triangles.
`cents`	Vectors of centres.
`areas`	Areas of triangles.

Author(s)

David Sterratt

`flipped.triangles.cart`
Determine indicies of triangles that are flipped

Description

In the projection of points onto the sphere, some triangles maybe flipped, i.e. in the wrong orientation. This functions determines which triangles are flipped by computing the vector pointing to the centre of each triangle and comparing this direction to vector product of two sides of the triangle.

Usage

```
flipped.triangles.cart(P, Tt, R)
```

Arguments

P	Points in Cartesian coordinates
Tt	Triangulation of points
R	Radius of sphere

Value

List containing:

flipped	Indicies of in rows of Tt of flipped triangles.
cents	Vectors of centres.
areas	Areas of triangles.

Author(s)

David Sterratt

---

fp — *Piecewise smooth function used in area penalty*

Description

Derivative of f

Usage

```
fp(x, x0)
```

Arguments

x	Main argument
x0	The cutoff parameter. Above this value the function is zero.

Value

The value of the function.

Author(s)

David Sterratt

`getDss` *Get transformed spherical coordinates of datapoints*

Description

Get spherical coordinates of datapoints.

Usage

```
getDss(r)
```

Arguments

r	`ReconstructedDataset` or `RetinalReconstructedDataset` object.

Value

`Dss`

Author(s)

David Sterratt

`getDss.reconstructedDataset` *Get transformed spherical coordinates of datapoints*

Description

Get spherical coordinates of datapoints.

Usage

```
## S3 method for class 'reconstructedDataset'
getDss(r)
```

Arguments

r	`ReconstructedDataset` object.

Value

`Dss`

Author(s)

David Sterratt

getDss.retinalReconstructedDataset
Get transformed spherical coordinates of datapoints

Description

Get spherical coordinates of datapoints, transformed according to the values of `DVflip` and `side`.

Usage

```
## S3 method for class 'retinalReconstructedDataset'
getDss(r)
```

Arguments

r	`RetinalReconstructedDataset` object.

Value

Dss

Author(s)

David Sterratt

getDssHullarea
Get area of convex hull around data points on sphere

Description

Get area of convex hull around data points on sphere

Usage

```
getDssHullarea(r)
```

Arguments

r	codeReconstructedDataset or `RetinalReconstructedDataset` object.

Value

Area in degress squared

Author(s)

David Sterratt

`getDssMean` *Karcher mean of datapoints in spherical coordinates*

Description

Get Karcher mean of datapoints in spherical coordinates.

Usage

```
getDssMean(r)
```

Arguments

`r`	`ReconstructedDataset` or `RetinalReconstructedDataset` object.

Value

`Dss.mean`

Author(s)

David Sterratt

`getDssMean.reconstructedDataset`
Karcher mean of datapoints in spherical coordinates

Description

Get Karcher mean of datapoints in spherical coordinates.

Usage

```
## S3 method for class 'reconstructedDataset'
getDssMean(r)
```

Arguments

`r`	`ReconstructedDataset` or `RetinalReconstructedDataset` object.

Value

`Dss.mean`

Author(s)

David Sterratt

getDssMean.retinalReconstructedDataset — *Get transformed spherical coordinates of Karcher mean of datapoints*

Description

Get Karcher mean of datapoints in spherical coordinates, transformed according to the values of `DVflip` and `side`.

Usage

```
## S3 method for class 'retinalReconstructedDataset'
getDssMean(r)
```

Arguments

r	`RetinalReconstructedDataset` object.

Value

`Dss.mean`

Author(s)

David Sterratt

getGss — *Get grouped variable with locations in spherical coordinates.*

Description

Get grouped variable with locations in spherical coordinates.

Usage

```
getGss(r)
```

Arguments

r	`ReconstructedDataset` or `RetinalReconstructedDataset` object.

Value

`Gss`

Author(s)

David Sterratt

getGss.reconstructedDataset

Get grouped variable with locations in spherical coordinates.

Description

Get grouped variable with locations in spherical coordinates.

Usage

```
## S3 method for class 'reconstructedDataset'
getGss(r)
```

Arguments

r	ReconstructedDataset object.

Value

Gss

Author(s)

David Sterratt

getGss.retinalReconstructedDataset

Get grouped variable with locations in spherical coordinates.

Description

Get grouped variable with locations in spherical coordinates.

Usage

```
## S3 method for class 'retinalReconstructedDataset'
getGss(r)
```

Arguments

r	ReconstructedDataset or RetinalReconstructedDataset object.

Value

Gss

Author(s)

David Sterratt

`getIDs` *Get IDs of groups of data within a dataset*

Description

Get IDs of groups of data within a dataset

Usage

```
getIDs(r)
```

Arguments

`r`	Object

`getIDs.dataset` *Get IDs of groups of data within a dataset*

Description

Get IDs of groups of data within a dataset

Usage

```
## S3 method for class 'dataset'
getIDs(r)
```

Arguments

`r`	`Dataset` object

Value

Array of IDs

Author(s)

David Sterratt

getIDs.reconstructedDataset

Get IDs of groups of data within a ReconstructedDataset

Description

Get IDs of groups of data within a ReconstructedDataset

Usage

```
## S3 method for class 'reconstructedDataset'
getIDs(r)
```

Arguments

r	ReconstructedDataset object

Value

Array of IDs

Author(s)

David Sterratt

getIms

Get coordinates of corners of pixels of image in spherical coordinates

Description

Get coordinates of corners of pixels of image in spherical coordinates

Usage

```
getIms(r)
```

Arguments

r	ReconstructedOutline or RetinalReconstructedOutline object

Value

Coordinates of corners of pixels in spherical coordinates

Author(s)

David Sterratt

getIms.reconstructedOutline

Get coordinates of corners of pixels of image in spherical coordinates

Description

Get coordinates of corners of pixels of image in spherical coordinates

Usage

```
## S3 method for class 'reconstructedOutline'
getIms(r)
```

Arguments

`r`	`ReconstructedOutline` object

Value

Coordinates of corners of pixels in spherical coordinates

Author(s)

David Sterratt

getKDE

Get kernel density estimate of data points

Description

Get kernel density estimate of data points

Usage

```
getKDE(r)
```

Arguments

`r`	`ReconstructedDataset` object

Value

See `compute.kernel.estimate`

Author(s)

David Sterratt

getKR — *Get kernel regression estimate of grouped data points*

Description

Get kernel regression estimate of grouped data points

Usage

```
getKR(r)
```

Arguments

r	ReconstructedDataset object

Value

See compute.kernel.estimate

Author(s)

David Sterratt

getSss — *Get transformed spherical coordinates of landmarks.*

Description

Get spherical coordinates of landmarks.

Usage

```
getSss(r)
```

Arguments

r	ReconstructedDataset or RetinalReconstructedDataset object.

Value

Sss

Author(s)

David Sterratt

getSss.reconstructedDataset
Get transformed spherical coordinates of landmarks.

Description

Get spherical coordinates of landmarks.

Usage

```
## S3 method for class 'reconstructedDataset'
getSss(r)
```

Arguments

r	ReconstructedDataset object.

Value

Sss

Author(s)

David Sterratt

getSss.retinalReconstructedDataset
Get transformed spherical coordinates of datapoints

Description

Get spherical coordinates of landmarks, transformed according to the values of DVflip and side.

Usage

```
## S3 method for class 'retinalReconstructedDataset'
getSss(r)
```

Arguments

r	RetinalReconstructedDataset object.

Value

Dss

Author(s)

David Sterratt

getStrains — *Return strains edges are under in spherical retina*

Description

This function returns information about how edges on the sphere have been deformed from their flat state.

Usage

```
getStrains(r)
```

Arguments

`r`	A `ReconstructedOutline` object

Value

A list containing two data frames `flat` and `spherical`. Each data frame contains for each edge in the flat or spherical meshes:

`L`	Length of the edge in the flat outline
`l`	Length of the corresponding edge on the sphere
`strain`	The strain of each connection
`logstrain`	The logarithmic strain of each connection

Author(s)

David Sterratt

getTear — *Return indicies of tear in AnnotatedOutline*

Description

Return indicies of tear in AnnotatedOutline

Usage

```
getTear(o, tid)
```

Arguments

`o`	`AnnotatedOutline` object
`tid`	Tear ID, which can be returned from `whichTear()`

Value

Vector of three point IDs, labelled with `V0`, `VF` and `VB`

Author(s)

David Sterratt

---

getTss.reconstructedOutline

Get spherical coordinates of tears.

Description

Get spherical coordinates of tears.

Usage

```
## S3 method for class 'reconstructedOutline'
getTss(r)
```

Arguments

r	`reconstructedOutline` object.

Value

`Tss`

Author(s)

David Sterratt

---

getTss.retinalReconstructedOutline

Get spherical coordinates of tears.

Description

This flips the tears according to whether the retina is DV flipped or not.

Usage

```
## S3 method for class 'retinalReconstructedOutline'
getTss(r)
```

Arguments

r	`RetinalReconstructedOutline` object.

Value

List of tears in spherical coordinates.

Author(s)

David Sterratt

`identity.transform` *The identity transformation*

Description

The identity transformation

Usage

```
identity.transform(r, ...)
```

Arguments

`r`	Coordinates of points in spherical coordinates represented as 2 column matrix with column names `phi` (lattitude) and `lambda` (longitude).
`...`	Other arguments

Value

Idendtical matrix

Author(s)

David Sterratt

`idt.read.dataset` *Read one of the Thompson lab's retinal datasets*

Description

Read one of the Thompson lab's retinal datasets. Each dataset is a folder containing a SYS file in SYSTAT format and a MAP file in text format. The SYS file specifies the locations of the data points and the MAP file specifies the outline.

Usage

```
idt.read.dataset(dataset, d.close = 0.25)
```

Arguments

`dataset`	Path to directory containing as SYS and MAP file
`d.close`	Maximum distance between points for them to count as the same point. This is expressed as a fraction of the width of the outline.

Details

The function returns the outline of the retina. In order to do so, it has to join up the segments of the MAP file. The tracings are not always precise; sometimes there are gaps between points that are actually the same point. The parameter `d.close` specifies how close points must be to count as the same point.

Value

`dataset`	The path to the directory given as an argument
`raw`	List containing<br>`map` The raw MAP data<br>`sys` The raw SYS data
`P`	The points of the outline
`gf`	Forward pointers along the outline
`gb`	Backward pointers along the outline
`Ds`	List of datapoints
`Ss`	List of landmark lines

Author(s)

David Sterratt

`ijroi.read.dataset` *Read a retinal dataset in IJROI format*

Description

Read a retinal dataset in IJROI format. Each dataset is a folder containing a file called `outline.roi` that specifies the outline in X-Y coordinates. It may also contain a file `datapoints.csv`, containing the locations of data points; see `read.datapoints` for the format of this file.

Usage

```
ijroi.read.dataset(dataset)
```

Arguments

`dataset`	Path to directory containing `outline.roi`

Value

A `RetinalDataset` object

Author(s)

David Sterratt

incident.to.lens.angle

Convert incident angle to angle on lens

Description

Convert incident angle to angle on lens

Usage

```
incident.to.lens.angle(psi, anp, S)
```

Arguments

psi	Incident angles
anp	Anterior nodal point on x-axis
S	Lens system

Value

Angles on lens

Author(s)

David Sterratt

Examples

```
S <- S.mouse.ss.23
draw.system(S, xoff=-2)
## imfile <- "eye23.png"
## im <- as.raster(readPNG(imfile))
scale <- 0.0086
xoff <- -0.52
yoff <- 0.31
## rasterImage(im, xoff, yoff-scale*nrow(im)/2, xoff+scale*ncol(im), yoff+scale*nrow(im)/
draw.system(S, add=TRUE, col="yellow")
anp <- 1
psi <- 0.1
psi <- 0.2
## Trace ray angles using nodal point at 0.2 - not too bad an approximation
## trace.ray.angle(0.9, 0.2, S)
## All that's needed is to get the angle at which the ray hits the final lens.
## Then we have a mapping from psi to phi.
## r <- trace.ray(c(-500, 101.5, -0.2), S)
angles <- seq(0, 90, by=10)
lens.angles <- 180/pi*incident.to.lens.angle(angles*pi/180, 0.2, S)
```

invert.sphere *Invert sphere about its centre*

Description

Invert sphere about its centre

Usage

```
invert.sphere(r, ...)
```

Arguments

r	Coordinates of points in spherical coordinates represented as 2 column matrix with column names phi (lattitude) and lambda (longitude).
...	Other arguments

Value

Matrix in same format, but with pi added to lambda and phi negated.

Author(s)

David Sterratt

invert.sphere.to.hemisphere *Invert sphere to hemisphere*

Description

Invert image of a partial sphere and scale the longitude so that points at lattitude phi0 is projected onto a longitude of 0 degrees (the equator).

Usage

```
invert.sphere.to.hemisphere(r, phi0, ...)
```

Arguments

r	Coordinates of points in spherical coordinates represented as 2 column matrix with column names phi (lattitude) and lambda (longitude).
phi0	The lattitude to map onto the equator
...	Other arguments

Value

Matrix in same format, but with pi added to lambda and phi negated and scaled so that the longitude phi0 is projected to 0 degrees (the equator)

Author(s)

David Sterratt

`karcher.mean.sphere` *Karcher mean on the sphere*

Description

The Karcher mean of a set of points on a manifold is defined as the point whose sum of squared Riemmann distances to the points is minimal. On a sphere using sphereical coordinates this distance can be computed using the formula for central angle.

Usage

```
karcher.mean.sphere(x, na.rm = FALSE, var = FALSE)
```

Arguments

`x`	Matrix of points on sphere as N-by-2 matrix with labelled columns \codephi (lattitude) and `lambda` (longitude)
`na.rm`	logical value indicating whether `NA` values should be stripped before the computation proceeds.
`var`	logical value indicating whether variance should be returned too.

Value

Vector of means with components named `phi` and `lambda`. If `var` is `TRUE`, a list containing mean and varience in elements `mean` and `var`.

Author(s)

David Sterratt

References

Heo, G. and Small, C. G. (2006). Form representations and means for landmarks: A survey and comparative study. *Computer Vision and Image Understanding*, 102:188-203.

See Also

`central.angle`

kde.compute.concentration *Find the optimal concentration for a set of data*

Description

Find the optimal concentration for a set of data

Usage

```
kde.compute.concentration(mu)
```

Arguments

mu	Data in spherical coordinates

Value

The optimal concentration

Author(s)

David Sterratt

kde.fhat *Kernel density estimate on sphere using Fisherian density with polar coordinates*

Description

Kernel density estimate on sphere using Fisherian density with polar coordinates

Usage

```
kde.fhat(r, mu, kappa)
```

Arguments

r	Locations at which to estimate density in polar coordinates
mu	Locations of data points in polar coordinates
kappa	Concentration parameter

Value

Vector of density estimates

Author(s)

David Sterratt

`kde.fhat.cart` *Kernel density estimate on sphere using Fisherian density with Cartesian coordinates*

Description

Kernel density estimate on sphere using Fisherian density with Cartesian coordinates

Usage

```
kde.fhat.cart(r, mu, kappa)
```

Arguments

`r`	Locations at which to estimate density in Cartesian coordinates on unit sphere
`mu`	Locations of data points in Cartesian coordinates on unit sphere
`kappa`	Concentration parameter

Value

Vector of density estimates

Author(s)

David Sterratt

`kde.L` *Estimate of the log likelihood of the points mu given a particular value of the concentration kappa*

Description

Estimate of the log likelihood of the points mu given a particular value of the concentration kappa

Usage

```
kde.L(mu, kappa)
```

Arguments

`mu`	Locations of data points in Cartesian coordinates on unit sphere
`kappa`	Concentration parameter

Value

Log likelihood of data

Author(s)

David Sterratt

`kr.compute.concentration` *Find the optimal concentration for a set of data*

Description

Find the optimal concentration for a set of data

Usage

```
kr.compute.concentration(mu, y)
```

Arguments

`mu`	Locations in Cartesian coordinates (independent variables)
`y`	Values at locations (dependent variables)

Value

The optimal concentration

Author(s)

David Sterratt

`kr.sscv` *Cross validation estimate of the least squares error of the points mu given a particular value of the concentration kappa*

Description

Cross validation estimate of the least squares error of the points mu given a particular value of the concentration kappa

Usage

```
kr.sscv(mu, y, kappa)
```

Arguments

`mu`	Locations in Cartesian coordinates (independent variables)
`y`	Values at locations (dependent variables)
`kappa`	Concentration parameter

Value

Least squares error

Author(s)

David Sterratt

`kr.yhat` *Kernel regression on sphere using Fisherian density with polar coordinates*

Description

Kernel regression on sphere using Fisherian density with polar coordinates

Usage

```
kr.yhat(r, mu, y, kappa)
```

Arguments

`r`	Locations at which to estimate dependent variables in polar coordinates
`mu`	Locations in polar coordinates (independent variables)
`y`	Values at data points (dependent variables)
`kappa`	Concentration parameter

Value

Estimates of dependent variables at locations `r`

Author(s)

David Sterratt

`kr.yhat.cart` *Kernel regression on sphere using Fisherian density with Cartesian coordinates*

Description

Kernel regression on sphere using Fisherian density with Cartesian coordinates

Usage

```
kr.yhat.cart(r, mu, y, kappa)
```

Arguments

`r`	Locations at which to estimate dependent variables in Cartesian coordinates
`mu`	Locations in Cartesian coordinates (independent variables)
`y`	Values at locations (dependent variables)
`kappa`	Concentration parameter

Value

Estimates of dependent variables at locations `r`

Author(s)

David Sterratt

`labelTearPoints` *Label three outline point indicies as apicies and vertices of tear*

Description

Label a set of three unlabelled points supposed to refer to the apex and vertcies of a cut and tear with the V0 (Apex), VF (forward vertex) and VB (backward vertex) labels.

Usage

```
labelTearPoints(o, m)
```

Arguments

`m`	the vector of three indicies
`o`	Outline object

Value

Vector of indicies labelled with V0, VF and VB

Author(s)

David Sterratt

`line.line.intersection`
Determine intersection between two lines

Description

Determine the intersection of two lines L1 and L2 in two dimensions, using the formula described by Weisstein.

Usage

```
line.line.intersection(P1, P2, P3, P4,
  interior.only = FALSE)
```

Arguments

`P1`	vector containing x,y coordinates of one end of L1
`P2`	vector containing x,y coordinates of other end of L1
`P3`	vector containing x,y coordinates of one end of L2
`P4`	vector containing x,y coordinates of other end of L2
`interior.only`	boolean flag indicating whether only intersections inside L1 and L2 should be returned.

Value

Vector containing x,y coordinates of intersection of L1 and L2. If L1 and L2 are parallel, this is infinite-valued. If `interior.only` is `TRUE`, then when the intersection does not occur between P1 and P2 and P3 and P4, a vector containing `NA`s is returned.

Author(s)

David Sterratt

Source

Weisstein, Eric W. "Line-Line Intersection." From MathWorld–A Wolfram Web Resource. http://mathworld.wolfram.com/Line-LineIntersection.html

Examples

```
## Intersection of two intersecting lines
line.line.intersection(c(0, 0), c(1, 1), c(0, 1), c(1, 0))

## Two lines that don't intersect
line.line.intersection(c(0, 0), c(0, 1), c(1, 0), c(1, 1))
```

`list.datasets` *List datasets underneath a directory*

Description

List valid datasets underneath a directory. This reports all directories that appear to be valid.

Usage

```
list.datasets(path = ".", verbose = FALSE)
```

Arguments

`path`	Directory path to start searching from
`verbose`	If `TRUE` report on progress

Value

A vector of directories containing datasets

Author(s)

David Sterratt

`lvsLplot.reconstructedOutline` *Plot the fractional change in length of mesh edges*

Description

Plot the fractional change in length of mesh edges. The length of each edge in the mesh in the reconstructed object is plotted against each edge in the spherical object. The points are colour-coded according to the amount of log strain each edge is under.

Usage

```
## S3 method for class 'reconstructedOutline'
lvsLplot(r)
```

Arguments

`r`	`reconstructedOutline` object

Author(s)

David Sterratt

`mergePointsEdges` *Merge stitched points and edges*

Description

This function creates merged and transformed versions (all suffixed with `t`) of a number of existing variables, as well as a matrix `Bt`, which maps a binary vector representation of edge indicies onto a binary vector representation of the indicies of the points linked by the edge.

Usage

```
mergePointsEdges(t)
```

Arguments

`t`	A `StitchedOutline` object in which points that have been added by stitching have been triangulated

Value

Adds following fields to input

`Pt`	Transformed point locations
`Tt`	Transformed triangulation
`Ct`	Transformed connection set
`Cut`	Transformed symmetric connection set

`Bt`	Transformed binary vector representation of edge indicies onto a binary vector representation of the indicies of the points linked by the edge
`Lt`	Transformed edge lengths
`ht`	Transformed correspondences
`u`	Indicies of unique points in untransformed space
`U`	Transformed indicies of unique points in untransformed space
`Rset`	The set of points on the rim (which has been reoorded)
`Rsett`	Transformed set of points on rim
`i0t`	Transformed index of the landmark
`H`	mapping from edges onto corresponding edges
`Ht`	Transformed mapping from edges onto corresponding edges

Author(s)

David Sterratt

`name.list` *Return a new version of the list in which any un-named elements have been given standardised names*

Description

Return a new version of the list in which any un-named elements have been given standardised names

Usage

```
name.list(l)
```

Arguments

`l`	the list with un-named elements

Value

The list with standardised names

Author(s)

David Sterratt

`nameLandmark.dataset` *Name a landmark in a Dataset*

Description

Name a landmark in a `dataset`. The name of element `i` of `Ss` is set to `name`, the name of any element that bore the name is set to "" and all other elements are unaltered.

Usage

```
## S3 method for class 'dataset'
nameLandmark(d, i, name)
```

Arguments

`d`	`dataset` object
`i`	index of landmark to name
`name`	name to give landmark

Value

New `dataset` object in which landmark is named

Author(s)

David Sterratt

`nameLandmark.retinalDataset` *Name a landmark in a Dataset*

Description

Name a landmark in a `dataset`. The name of element `i` of `Ss` is set to `name`, the name of any element that bore the name is set to "" and all other elements are unaltered.

Usage

```
## S3 method for class 'retinalDataset'
nameLandmark(d, i, name)
```

Arguments

`d`	`dataset` object
`i`	index of landmark to name
`name`	name to give landmark

Value

New `dataset` object in which landmark is named

Author(s)

David Sterratt

`new.ray` *Compute origin and deflection of new ray*

Description

Compute origin and deflection of new ray

Usage

```
new.ray(r0, S)
```

Arguments

`r0`	Vector comprising Cartesian coordinates of incident ray origin, angle of incident ray with x-axis and index of system surface that origin lies on, `NA` if the origin is not associated with any surface.
`S`	S System defined by data frame containing left margin of surfaces (A), radii of surfaces (R), cut-offs (C) and refracive indices (n).

Value

Vector comprinsing new origin and angle

Author(s)

David Sterratt

`normalise.angle` *Bring angle into range*

Description

Bring angle into range

Usage

```
normalise.angle(theta)
```

Arguments

`theta`	Angle to bring into range `[-pi, pi]`

Value

Normalised angle

Author(s)

David Sterratt

`optimiseMapping` *Optimise mapping*

Description

Optimise the mapping from the flat outline to the sphere

Usage

```
optimiseMapping(r, alpha = 4, x0 = 0.5, nu = 1,
  method = "BFGS", plot.3d = FALSE, dev.flat = NA,
  dev.polar = NA, control = list())
```

Arguments

`r`	reconstructedOutline object
`alpha`	Area penalty scaling coefficient
`x0`	Area penalty cutoff coefficient
`nu`	Power to which to raise area
`method`	Method to pass to `optim`
`plot.3d`	If `TRUE` make a 3D plot in an RGL window
`dev.flat`	Device handle for plotting grid to
`dev.polar`	Device handle for plotting ploar plot to
`control`	Control arguent to passt `optim`

Value

reconstructedOutline object

Author(s)

David Sterratt

`orthographic` *Orthographic projection*

Description

Orthographic projection

Usage

```
orthographic(r, proj.centre = cbind(phi = 0, lambda = 0),
  ...)
```

Arguments

`r`	Lattitude-longitude coordinates in a matrix with columns labelled `phi` (lattitude) and `lambda` (longitude)
`proj.centre`	Location of centre of projection as matrix with column names `phi` (elevation) and `lambda` (longitude).
`...`	Arguments not used by this projectio.n

Value

Two-column matrix with columns labelled `x` and `y` of locations of projection of coordinates on plane

Author(s)

David Sterratt

References

http://en.wikipedia.org/wiki/Map_projection, http://mathworld.wolfram.com/OrthographicProjection.html

---

`Outline` *Outline constructor*

Description

Construct an outline object. This sanitises the input points `P`, as described below.

Usage

```
Outline(P, scale = NA, im = NULL)
```

Arguments

`P`	The points of the outline. The last point is not repeated.
`scale`	The length of one unit of `P` in micrometres. When images are present, this is the length of the side of a pixel in the image.
`im`	An image as a `raster` object

Value

An `Outline` object containing the following:

`P`	A N-by-2 matrix of points of the `Outline` arranged in anticlockwise order
`gf`	For each row of `P`, the index of `P` that is next in the outline travelling anticlockwise (forwards)
`gb`	For each row of `P`, the index of `P` that is next in the outline travelling clockwise (backwards)
`im`	The image as a `raster` object
`scale`	The length of one unit of `P` in micrometres

Author(s)

David Sterratt

`panlabel` *Ancillary function to place labels*

Description

Ancillary function to place labels

Usage

```
panlabel(panlabel, line = -0.7)
```

Arguments

`panlabel`	Label text
`line`	Line on which to appear

Author(s)

David Sterratt

`plot.IJROI` *Plot IJROI object*

Description

Plot IJROI object

Usage

```
## S3 method for class 'IJROI'
plot(x, add = FALSE, ...)
```

Arguments

`x`	The IJROI object
`add`	Whether to add to an existing plot
`...`	Additional parameters

Author(s)

David Sterratt

`polar.cart.to.sphere.spherical`
Convert polar projection in Cartesian coordinates to spherical coordinates on sphere

---

Description

This is the inverse of `sphere.spherical.to.polar.cart`

Usage

```
polar.cart.to.sphere.spherical(r, pa = FALSE,
  preserve = "lattitude")
```

Arguments

`r`	2-column Matrix of Cartesian coordinates of points on polar projection. Column names should be `x` and `y`
`pa`	If `TRUE`, make this an area-preserving projection
`preserve`	Quantity to preserve locally in the projection. Options are `lattitude`, `area` or `angle`

Value

2-column Matrix of spherical coordinates of points on sphere. Column names are `phi` and `lambda`.

Author(s)

David Sterratt

polartext — *Put text on the polar plot*

Description

Place text at bottom right of projection

Usage

```
polartext(text)
```

Arguments

text	Test to place

Author(s)

David Sterratt

projection — *Plot projection of an object*

Description

Generic function for plotting projections of objects.

Usage

```
projection(r, ...)
```

Arguments

r	Object such as a ReconstructedOutline
...	Other parameters; see projection.reconstructedOutline and projection.reconst[illegible]

Author(s)

David Sterratt

projection.reconstructedDataset
Plot projection of reconstructed dataset

Description

Plot projection of reconstructed dataset

Usage

```
## S3 method for class 'reconstructedDataset'
projection(r,
   transform = identity.transform,
   axisdir = cbind(phi = 90, lambda = 0),
   projection = azimuthal.equalarea,
   proj.centre = cbind(phi = 0, lambda = 0),
   lambdalim = c(-180, 180), datapoints = TRUE,
   datapoint.means = TRUE, datapoint.contours = TRUE,
   grouped = FALSE, grouped.contours = FALSE,
   landmarks = TRUE, ids = getIDs(r), ...)
```

Arguments

`r`	`ReconstructedDataset` object
`transform`	Transform function to apply to spherical coordinates before rotation
`axisdir`	Direction of axis (North pole) of sphere in external space
`projection`	Projection in which to display object, e.g. `azimuthal.equalarea` or `sinusoidal`
`proj.centre`	Location of centre of projection as matrix with column names `phi` (elevation) and `lambda` (longitude).
`lambdalim`	Limits of longitude (in degrees) to display
`datapoints`	If `TRUE`, display data points
`datapoint.means`	If `TRUE`, display Karcher mean of data points.
`datapoint.contours`	If `TRUE`, display contours around the data points generated using Kernel Density Estimation.
`grouped`	If `TRUE`, dipslay grouped data.
`grouped.contours`	If `TRUE`, display contours around the grouped data generated using Kernel Regression.
`landmarks`	If `TRUE`, dipslay landmarks.
`ids`	IDs of groups of data within a dataset, returned using `getIDs`.
`...`	Graphical parameters to pass to plotting functions

Author(s)

David Sterratt

---

projection.reconstructedOutline
Projection of a reconstructed outline

Description

Draw a projection of a ReconstructedOutline. This method sets up the grid lines and the angular labels and draws the image.

Usage

```
## S3 method for class 'reconstructedOutline'
projection(r,
   transform = identity.transform,
   axisdir = cbind(phi = 90, lambda = 0),
   projection = azimuthal.equalarea,
   proj.centre = cbind(phi = 0, lambda = 0),
   lambdalim = c(-180, 180), philim = c(-90, 90),
   labels = c(0, 90, 180, 270), grid = TRUE,
   grid.bg = "transparent", grid.int.minor = 15,
   grid.int.major = 45, colatitude = TRUE, pole = FALSE,
   image = TRUE, add = FALSE, ...)
```

Arguments

`r`	`ReconstructedOutline` object
`transform`	Transform function to apply to spherical coordinates before rotation
`axisdir`	Direction of axis (North pole) of sphere in external space as matrix with column names `phi` (elevation) and `lambda` (longitude).
`projection`	Projection in which to display object, e.g. `azimuthal.equalarea` or `sinusoidal`
`proj.centre`	Location of centre of projection as matrix with column names `phi` (elevation) and `lambda` (longitude).
`lambdalim`	Limits of longitude (in degrees) to display
`philim`	Limits of latitude (in degrees) to display
`labels`	Vector of 4 labels to plot at 0, 90, 180 and 270 degrees
`grid`	Whether or not to show the grid lines of lattitude and longitude
`grid.bg`	Background colour of the grid
`grid.int.minor`	Interval between minor grid lines in degrees
`grid.int.major`	Interval between major grid lines in degrees
`colatitude`	If `TRUE` have radial labels plotted with respect to colatitude rather than latitude
`pole`	If `TRUE` indicate the pole with a "*"
`image`	If `TRUE`, show the image
`add`	If `TRUE`, don't draw axes; add to existing plot.
`...`	Graphical parameters to pass to plotting functions

`projectToSphere` *Project mesh points in the flat outline onto a sphere*

Description

This takes the mesh points from the flat outline and maps them to the curtailed sphere. It uses the area of the flat outline and `phi0` to determine the radius `R` of the sphere. It tries to get a good first approximation by using the function `stretchMesh`.

Usage

```
projectToSphere(r)
```

Arguments

`r`	`Outline` object to which the following information has been added with `mergePointsEdges`: `Pt` The mesh point coordinates. `Rsett` The set of points on the rim. `A.tot` The area of the flat outline.

Value

`reconstructedOutline` object containing the following extra information

`phi`	Lattitude of mesh points.
`lmabda`	Longitude of mesh points.
`R`	Radius of sphere.

Author(s)

David Sterratt

`ray.arc.intersection` *Compute intersection of ray and arc*

Description

Compute intersection of ray and arc

Usage

```
ray.arc.intersection(Ptheta, A, R, C)
```

Arguments

`Ptheta`	Vector comprising cartesian coordinates of incident ray origin and angle with x-axis of incident ray
`A`	Left-edge of circle on x-axis
`R`	Radius of circle
`C`	Angle at which arc cut off

Value

Vector of x, y and lambda

Author(s)

David Sterratt

`Rcart` *Restore points to spherical manifold*

Description

Restore points to spherical manifold after an update of the Lagrange integration rule

Usage

```
Rcart(P, R, Rset, i0, phi0, lambda0)
```

Arguments

`P`	Point positions as N-by-3 matrix
`R`	Radius of sphere
`Rset`	Indicies of points on rim
`i0`	Index of fixed point
`phi0`	Cutoff of curtailed sphere in radians
`lambda0`	Longitude of fixed point on rim

Value

Points projected back onto sphere

Author(s)

David Sterratt

`read.datapoints` *Read data points in CSV format*

Description

Read data points from a file `datapont s.csv` in the directory `dataset`. The CSV should contain two columns for every dataset. Each pair of columns must contain a unique name in the first cell of the first row and a valid colour in the second cell of the first row. In the remaining rows, the X coordinates of data points should be in the first column and the Y coordinates should be in the second column.

Usage

```
read.datapoints(dataset)
```

Arguments

`dataset`	Path to directory containing `datapont s.csv`

Value

List containing

`Ds`	List of sets of datapoints. Each set comprises a 2-column matrix and each set is named.
`cols`	List of colours for each dataset. There is one element that corresponds to each element of `Ds` and which bears the same name.

Author(s)

David Sterratt

`read.ijroi` *Read an ImageJ ROI file*

Description

Read an ImageJ ROI file. This returns a structure containing the ImageJ data.

Usage

```
read.ijroi(file, verbose = FALSE)
```

Arguments

`file`	Name of ImageJ ROI file to read
`verbose`	Whether to report information

Value

A structure of class IJROI containing the ROI information

Author(s)

David Sterratt

Examples

```
path <- file.path(system.file(package = "retistruct"), "extdata", "ijroi")
im <- as.raster(readPNG(file.path(path, "imagej-logo.png")))
plot(NA, NA, xlim=c(0, ncol(im)), ylim=c(nrow(im), 0), asp=1)
rasterImage(im, 0,  nrow(im), ncol(im), 0, interpolate=FALSE)
r <- read.ijroi(file.path(path, "rect.roi"))
plot(r, TRUE)
r <- read.ijroi(file.path(path, "polygon.roi"))
plot(r, TRUE)
r <- read.ijroi(file.path(path, "oval.roi"))
plot(r, TRUE)
```

`ReconstructedDataset` *Constructor for RecontructedDataset object*

Description

This function infers the coordinates of datapoints `Ds` and landmarks `Ss` in spherical coordinates.

Usage

```
ReconstructedDataset(r, report = message)
```

Arguments

`r`	Object that of clases `reconstructedOutline` and `dataset`.
`report`	Function used to report progress.

Value

`ReconstructedDataset` object containing the input information and the following modified and extra information:

`Dsb`	Datapoints in barycentric coordinates
`Dsc`	Datapoints on reconstructed sphere in cartesian coordinates
`Dss`	Datapoints on reconstructed sphere in spherical coordinates
`Ssb`	Landmarks in barycentric coordinates
`Ssc`	Landmarks on reconstructed sphere in cartesian coordinates
`Sss`	Landmarks on reconstructed sphere in spherical coordinates

Author(s)

David Sterratt

ReconstructedOutline *Reconstruct outline into spherical surface*

Description

Reconstruct outline into spherical surface. Reconstruction proceeds in a number of stages:

Usage

```
ReconstructedOutline(o, n = 500, alpha = 8, x0 = 0.5,
  report = print, plot.3d = FALSE, dev.flat = NA,
  dev.polar = NA)
```

Arguments

`o`	`AnnotatedOutline` object, containing the following information:<br>`P` outline points as N-by-2 matrix<br>`V0` indicies of the apex of each tear<br>`VF` indicies of the forward vertex of each tear<br>`VB` indicies of the backward vertex of each tear<br>`i0` index of the landmark on the rim<br>`phi0` lattitude of rim of partial sphere<br>`lambda0` longitude of landmark on rim
`n`	Number of points in triangulation.
`alpha`	Area scaling coefficient
`x0`	Area cutoff coefficient
`report`	Function used to report progress.
`plot.3d`	Whether to show 3D picture during optimisation.
`dev.flat`	Device to plot grid onto. Value of `NA` (default) means no plotting.
`dev.polar`	Device display projection. Value of NA (default) means no plotting.

Details

1. The flat object is triangulated with at least `n` triangles. This can introduce new vertices in the rim.
2. The triangulated object is stitched.
3. The stitched object is triangulated again, but this time it is not permitted to add extra vertices to the rim.
4. The corresponding points determined by the stitching process are merged to form a new set of merged points and a new triangulation.
5. The merged points are projected roughly to a sphere.
6. The locations of the points on the sphere are moved so as to minimise the energy function.

Value

`reconstructedOutline` object containing the input information and the following modified and extra information:

`P`	New set of points in flattened object
`gf`	New set of forward pointers in flattened object
`gb`	New set of backward pointers in flattened object
`phi`	lattitude of new points on sphere
`lambda`	longitude of new points on sphere
`Tt`	New triangulation

Author(s)

David Sterratt

`remove.identical.consecutive.rows` *Remove identical consecutive rows from a matrix*

Description

This is simlar to unique(), but spares rows which are duplicated, but at different points in the matrix

Usage

```
remove.identical.consecutive.rows(P)
```

Arguments

`P`	Source matrix

Value

Matrix with identical consecutive rows removed.

Author(s)

David Sterratt

remove.intersections

Remove intersections between adjacent segements in a closed path

Description

Suppose segments AB and CD intersect. Point B is replaced by the intersection point, defined B'. Point C is replaced by a point C' on the line B'D. The maxium distance of B'C' is given by the parameter d. If the distance l B'D is less than 2d, the distance B'C' is l/2.

Usage

```
remove.intersections(P, d = 50)
```

Arguments

`P`	The points, as a 2-column matrix
`d`	Criterion for maximum distance when points are inser

Value

A new closed path without intersections

Author(s)

David Sterratt

removeTear

Remove tear from an AnnotatedOutline

Description

Remove tear from an AnnotatedOutline

Usage

```
removeTear(o, tid)
```

Arguments

`o`	`AnnotatedOutline` object
`tid`	Tear ID, which can be returned from `whichTear()`

Value

`AnnotatedOutline` object

Author(s)

David Sterratt

RetinalDataset *RetinalDataset constructor*

Description

Construct an RetinalDataset that contains information specific to the dataset in question.

Usage

```
RetinalDataset(d)
```

Arguments

d	A dataset object

Value

An retinalDataset object. This contains all the information from d plus:

DVflip	TRUE if the raw data is flipped in the dorsoventral direction
side	The side of the eye ("Left" or "Right")

Author(s)

David Sterratt

RetinalReconstructedDataset *RetinalReconstructedDataset constructor*

Description

Create an object that is specific to retinal datasets. This contains methods that return datapoint and landmark coordinates that have been transformed according to the values of DVflip and side.

Usage

```
RetinalReconstructedDataset(r, report = message)
```

Arguments

r	Object that inherits both reconstructedDataset and dataset.
report	Function used to report progress.

Value

RetinalReconstructedDataset object. This does not contain any extra fields, but there are extra mthods dthat apply to it.

Author(s)

David Sterratt

`RetinalReconstructedOutline` *RetinalReconstructedOutline constructor*

Description

Create an object that is specific to retinal datasets. This contains methods that return datapoint and landmark coordinates that have been transformed according to the values of `DVflip` and `side`.

Usage

```
RetinalReconstructedOutline(r, report = message)
```

Arguments

`r`	Object that inherits `ReconstructedOutline`
`report`	Function used to report progress.

Value

`RetinalReconstructedOutline` object. This does not contain any extra fields, but there are extra mthods dthat apply to it.

Author(s)

David Sterratt

`retistruct.batch` *Batch operation using the multicore package*

Description

This function reconstructs a number of datasets, using the R `multicore` package to distribute the reconstruction of multiple datasets across CPUs. If `datasets` is not specified the function recurses through a directory tree starting at `tldir`, determining whether the directory contains valid raw data and markup, and performing the reconstruction if it does.

Usage

```
retistruct.batch(tldir = ".", outputdir = tldir,
  datasets = NULL, device = "pdf", titrate = FALSE,
  cpu.time.limit = 3600, mc.cores = getOption("cores"))
```

Arguments

`tldir`	If datasets is not specified, the top level of the directory tree through which to recurse in order to find datasets.
`outputdir`	directory in which to dump a log file and images
`datasets`	Vector of dataset directories to reconstruct
`device`	string indicating what type of graphics output required. Options are "pdf" and "png".
`titrate`	Whether to "titrate" the reconstruction for different values of `phi0`. See `titrate.reconstructe`
`cpu.time.limit`	amount of CPU after which to terminate the process
`mc.cores`	The number of cores to use. Defaults to the total number available.

Author(s)

David Sterratt

`retistruct.batch.analyse.summaries`
Extract statistics from a directory containing reconstruction directories.

Description

Extract statistics from a directory containing reconstruction directories.

Usage

```
retistruct.batch.analyse.summaries(path)
```

Arguments

`path`	Directory containing recontstruction directories

Value

Data frame containg various statistics

Author(s)

David Sterratt

`retistruct.batch.analyse.summary`
Extract statistics from the retistruct-batch.csv summary file

---

Description

Extract statistics from the retistruct-batch.csv summary file

Usage

```
retistruct.batch.analyse.summary(path)
```

Arguments

`path`	The path to the retistruct-batch.csv

Value

list of various statistics

Author(s)

David Sterratt

`retistruct.batch.export.matlab`
Export data from reconstruction data files to matlab

Description

Recurse through a directory tree, determining whether the directory contains valid derived data and converting r.rData files to files in matlab format named r.mat

Usage

```
retistruct.batch.export.matlab(tldir = ".")
```

Arguments

`tldir`	The top level of the directory tree through which to recurse

Author(s)

David Sterratt

retistruct.batch.figures

Plot figures for a batch of reconstructions

Description

Recurse through a directory tree, determining whether the directory contains valid derived data and plotting graphs if it does.

Usage

```
retistruct.batch.figures(tldir = ".", outputdir = tldir,
  ...)
```

Arguments

`tldir`	The top level directory of the tree through which to recurse.
`outputdir`	Directory in which to dump a log file and images
`...`	Parameters passed to plotting functions

Author(s)

David Sterratt

retistruct.batch.plot.ods

Superposed plot of ODs on polar axes

Description

Polar plot of ODs of a group of retinae.

Usage

```
retistruct.batch.plot.ods(summ, phi0d, ...)
```

Arguments

`summ`	Summary object returned by `retistruct.batch.summary`
`phi0d`	The rim angle for the plot
`...`	Other parameters, passed to projection

Value

A pseudo retina, in which the optic disks are treated as datapoints

Author(s)

David Sterratt

retistruct.batch.summary

Extract summary data for a batch of reconstructions

Description

Recurse through a directory tree, determining whether the directory contains valid derived data and extracting summary data if it does.

Usage

```
retistruct.batch.summary(tldir = ".", cache = TRUE)
```

Arguments

`tldir`	The top level directory of the tree through which to recurse.
`cache`	If `TRUE` use the cached statistics rather than generate on the fly (which is slower).

Value

Data frame containing summary data

Author(s)

David Sterratt

retistruct.check.markup

Retistruct check markup

Description

Check that markup such as tears and the nasal or dorsal points are present.

Usage

```
retistruct.check.markup(o)
```

Arguments

`o`	Outline object

Value

If all markup is present, return `TRUE`. Otherwise return `FALSE`.

Author(s)

David Sterratt

retistruct.cli — *Process a dataset with a time limit*

Description

This calls retistruct.cli.process with a time limit specified by cpu.time.limit.

Usage

```
retistruct.cli(dataset, cpu.time.limit = Inf,
  outputdir = NA, device = "pdf", ...)
```

Arguments

dataset	Path to dataset to process
cpu.time.limit	Time limit in seconds
outputdir	Directory in which to save any figures
device	String representing device to print figures to
...	Other arguments to pass to retistruct.cli.process

Value

A list comprising

status	0 for success, 1 for reaching cpu.time.limit and 2 for an unknown error
time	The time take in seconds
mess	Any error message

Author(s)

David Sterratt

retistruct.cli.figure — *Print a figure to file*

Description

Print a figure to file

Usage

```
retistruct.cli.figure(dataset, outputdir, device = "pdf",
  width = 6, height = 6, res = 100)
```

Arguments

`dataset`	Path to dataset to process
`outputdir`	Directory in which to save any figures
`device`	String representing device to print figures to
`width`	Width of figures in inches
`height`	Height of figures in inches
`res`	Resolution of figures in dpi (only applies to bitmap devices)

Author(s)

David Sterratt

`retistruct.cli.process` *Process a dataset, saving results to disk*

Description

This function processes a `dataset`, saving the reconstruction data and matlab export data to the `dataset` directory and printing figures to `outputdir`.

Usage

```
retistruct.cli.process(dataset, outputdir = NA,
  device = "pdf", titrate = FALSE)
```

Arguments

`dataset`	Path to dataset to process
`outputdir`	Directory in which to save any figures
`device`	String representing device to print figures to
`titrate`	If `TRUE` add output of `titrate.reconstructedOutline` to object saved.

Author(s)

David Sterratt

`retistruct.export.matlab` *Save reconstruction data in MATLAB format*

Description

Save as a MATLAB object certain fields of an object `r` that inherits `ReconstructedDataset` and `ReconstructedOutline` to a file called `r.mat` in the directory `r$dataset`.

Usage

```
retistruct.export.matlab(r)
```

Arguments

`r`	`ReconstructedDataset` object

Author(s)

David Sterratt

`retistruct.potential.od` *Test for a potential optic disc*

Description

Test the oputline object `o` for the prescense of potential optice disc. This is done by checking that the list of landmark lines `Ss` exists.

Usage

```
retistruct.potential.od(o)
```

Arguments

`o`	Outline object

Value

`TRUE` if an optic disc may be present; `FALSE` otherwise

Author(s)

David Sterratt

`retistruct.read.dataset` *Read a retinal dataset*

Description

Read a retinal dataset in one of three formats; for information on formats see see `idt.read.dataset`, `csv.read.dataset` and `ijroi.read.dataset`. The format is autodetected from the files in the directory.

Usage

```
retistruct.read.dataset(dataset, ...)
```

Arguments

`dataset`	Path to directory containing as SYS and MAP file
`...`	Parameters passed to the format-specific functions.

Value

An object that of classes `RetinalDataset` and `RetinalDataset`. There may be extra fields too, depending on the format.

Author(s)

David Sterratt

`retistruct.read.markup` *Read the markup data*

Description

Read the markup data contained in the files '`markup.csv`', '`P.csv`' and '`T.csv`' in the directory '`dataset`', which is specified in the reconstruction object `r`.

Usage

```
retistruct.read.markup(a, error = stop)
```

Arguments

`a`	Dataset object, containing `dataset` path
`error`	Function to run on error, by default `stop()`

Details

The tear information is contained in the files '`P.csv`' and '`T.csv`'. The first file contains the locations of outline points that the tears were marked up on. The second file contains the indicies of the apicies and backward and forward verticies of each tear. It is necessary to have the file of points just in case the algorithm that determines `P` in `retistruct.read.dataset` has changed since the markup of the tears.

The remaining information is contained containted in the file '`markup.csv`'.

If `DVflip` is specified, the locations of points `P` flipped in the y-direction. This operation also requires the swapping of `gf` and `gb` and `VF` and `VB`.

Value

o `RetinalDataset` object

`V0`	Indicies in `P` of apicies of tears
`VB`	Indicies in `P` of backward verticies of tears
`VF`	Indicies in `P` of backward verticies of tears
`iN`	Index in `P` of nasal point, or `NA` if not marked
`iD`	Index in `P` of dorsal point, or `NA` if not marked
`iOD`	Index in `Ss` of optic disc
`phi0`	Angle of rim in degrees
`DVflip`	Boolean variable indicating if DV axis has been flipped

Author(s)

David Sterratt

`retistruct.read.recdata` *Read the reconstruction data from file*

Description

Given an outline object with a `dataset` field, read the reconstruction data from the file '*dataset*`/r.Rdata`'.

Usage

```
retistruct.read.recdata(o, check = TRUE)
```

Arguments

`o`	Outline object containing `dataset` field
`check`	If `TRUE` check that the base information in the reconstruction object is the same as the base data in source files.

Value

If the reconstruction data exists, return a reconstruction object, else return the outline object `o`.

Author(s)

David Sterratt

`retistruct.reconstruct` *Reconstruct a retina*

Description

Reconstruct a retina

Usage

```
retistruct.reconstruct(o, report = retistruct.report,
  plot.3d = FALSE, dev.flat = NA, dev.polar = NA, ...)
```

Arguments

`o`	`AnnotatedOutline` object
`report`	Function to report progress
`plot.3d`	If `TRUE` show progress in a 3D plot
`dev.flat`	The ID of the device to which to plot the flat representation
`dev.polar`	The ID of the device to which to plot the polar representation
`...`	Parameters to be passed to `ReconstructedOutline`

Value

Object of classes `RetinalReconstructedOutline` and `RetinalReconstructedDataset` that contains all the reconstruction information

Author(s)

David Sterratt

`retistruct.save.markup` *Save markup*

Description

Save the makrup in the `RetinalDataset` `a` to a file called `markup.csv` in the directory `a$dataset`.

Usage

```
retistruct.save.markup(a)
```

Arguments

a	RetinalDataset object

Author(s)

David Sterratt

---

retistruct.save.recdata — *Save reconstruction data*

Description

Save the reconstruction data in an object r that inherits ReconstructedDataset and ReconstructedOutline to a file called r.Rdata in the directory r$dataset.

Usage

```
retistruct.save.recdata(r)
```

Arguments

r	ReconstructedDataset object

Author(s)

David Sterratt

---

rotate.axis — *Rotate axis of sphere*

Description

This rotates points on sphere by specifying the direction its polar axis, i.e. the axis going through (90, 0), should point after (a) a rotation about an axis through the poins (0, 0) and (0, 180) and (b) rotation about the original polar axis.

Usage

```
rotate.axis(r, r0)
```

Arguments

r	Coordinates of points in spherical coordinates represented as 2 column matrix with column names phi (lattitude) and lambda (longitude).
r0	Direction of the polar axis of the sphere on which to project represented as a 2 column matrix of with column names phi (lattitude) and lambda (longitude).

Value

2-column matrix of spherical coordinates of points with column names `phi` (lattidude) and `lambda` (longitude).

Author(s)

David Sterratt

Examples

```
r0 <- cbind(phi=0, lambda=-pi/2)
r <- rbind(r0, r0+c(1,0), r0-c(1,0), r0+c(0,1), r0-c(0,1))
r <- cbind(phi=pi/2, lambda=0)
rotate.axis(r, r0)
```

`setFixedPoint` *Set fixed point*

Description

Set fixed point

Usage

```
setFixedPoint(o, i0, name)
```

Arguments

`o`	`AnnotatedOutline` object
`i0`	Index of fixed point
`name`	Name of fixed point

Value

New `AnnotatedOutline` object

Author(s)

David Sterratt

`simplify.outline` *Simplify an outline object by removing short edges*

Description

Simplify an outline object by removing verticies bordering short edges while not encroaching on any of the outline. At present, this is done by finding concave vertices. It is safe to remove these, at the expense of increasing the area a bit.

Usage

```
simplify.outline(o, min.frac.length = 0.001,
  plot = FALSE)
```

Arguments

`o`	`outline` object to simplify
`min.frac.length`	the minumum length as a fraction of the total length of the outline.
`plot`	whether to display plotting or not during simplification

Value

Simlified `outline` object

Author(s)

David Sterratt

`sinusoidal` *Sinusoidal projection*

Description

Sinusoidal projection

Usage

```
sinusoidal(r, proj.centre = cbind(phi = 0, lambda = 0),
  lambdalim = NULL, lines = FALSE, ...)
```

Arguments

`r`	Lattitude-longitude coordinates in a matrix with columns labelled `phi` (latitude) and `lambda` (longitude). Alternatively string "boundary", indicating that boundary of projection should be drawn.
`proj.centre`	Location of centre of projection as matrix with column names `phi` (elevation) and `lambda` (longitude). Currently only longitude is used by this function.
`lambdalim`	Limits of longitude to plot
`lines`	If this is `TRUE` create breaks of `NA`s when lines cross the limits of longitude. This prevents lines crossing the centre of the projection.
`...`	Arguments not used by this projection.

Value

Two-column matrix with columns labelled `x` and `y` of locations of projection of coordinates on plane

Author(s)

David Sterratt

References

http://en.wikipedia.org/wiki/Map_projection, http://mathworld.wolfram.com/SinusoidalProjection.html

---

`solveMappingCart` *Optimise mapping*

Description

Optimise the mapping from the flat outline to the sphere

Usage

```
solveMappingCart(r, alpha = 4, x0 = 0.5, nu = 1,
  method = "BFGS", plot.3d = FALSE, dev.flat = NA,
  dev.polar = NA, ...)
```

Arguments

`r`	reconstructedOutline object
`alpha`	Area penalty scaling coefficient
`x0`	Area penalty cutoff coefficient
`nu`	Power to which to raise area
`method`	Method to pass to `optim`
`plot.3d`	If `TRUE` make a 3D plot in an RGL window
`dev.flat`	Device handle for plotting grid to
`dev.polar`	Device handle for plotting ploar plot to
`...`	Extra arguments to pass to `fire`

Value

reconstructedOutline object

Author(s)

David Sterratt

`sphere.cart.to.sphere.spherical`
Convert from Cartesian to spherical coordinates

Description

Convert locations on the surface of a sphere in cartesian (X, Y, Z) coordinates to spherical (phi, lambda) coordinates.

Usage

```
sphere.cart.to.sphere.spherical(P, R = 1)
```

Arguments

`P`	locations of points on sphere as N-by-3 matrix with labelled columns "X", "Y" and "Z"
`R`	radius of sphere

Details

It is assumed that all points are lying on the surface of a sphere of radius R.

Value

N-by-2 Matrix wtih columns ("phi" and "lambda") of locations of points in spherical coordinates

Author(s)

David Sterratt

`sphere.spherical.to.polar.cart`
Convert spherical coordinates on sphere to polar projection in Cartesian coordinates

---

Description

This is the inverse of `polar.cart.to.sphere.spherical`

Usage

```
sphere.spherical.to.polar.cart(r, pa = FALSE,
  preserve = "lattitude")
```

Arguments

`r`	2-column Matrix of spherical coordinates of points on sphere. Column names are `phi` and `lambda`.
`pa`	If `TRUE`, make this an area-preserving projection
`preserve`	Quantity to preserve locally in the projection. Options are `lattitude`, `area` or `angle`

Value

2-column Matrix of Cartesian coordinates of points on polar projection. Column names should be `x` and `y`

Author(s)

David Sterratt

`sphere.spherical.to.sphere.cart`
Convert from spherical to Cartesian coordinates

Description

Convert locations of points on sphere in spherical coordinates to points in 3D cartesian space

Usage

```
sphere.spherical.to.sphere.cart(phi, lambda, R = 1)
```

Arguments

`phi`	vector of lattitudes of N points
`lambda`	vector of longitudes of N points
`R`	radius of sphere

Value

An N-by-3 matrix in which each row is the cartesian (X, Y, Z) coordinates of each point

Author(s)

David Sterratt

`sphere.tri.area` *Area of triangles on a sphere*

Description

This uses l'Hullier's theorem to compute the spherical excess and hence the area of the spherical triangle.

Usage

```
sphere.tri.area(P, Pt)
```

Arguments

`P`	2-column matrix of vertices of triangles given in spherical polar coordinates. Columns need to be labelled `phi` (lattitude) and `lambda` (longitude).
`Pt`	3-column matrix of indicies of rows of `P` giving triangulation

Value

Vectors of areas of triangles in units of steradians

Author(s)

David Sterratt

Source

Wolfram MathWorld http://mathworld.wolfram.com/SphericalTriangle.html and http://mathworld.wolfram.com/SphericalExcess.html

Examples

```
## Something that should be an eighth of a sphere, i.e. pi/2
P <- cbind(phi=c(0, 0, pi/2), lambda=c(0, pi/2, pi/2))
Pt <- cbind(1, 2, 3)
## The result of this should be 0.5
print(sphere.tri.area(P, Pt)/pi)

## Now a small triangle
P1 <- cbind(phi=c(0, 0, 0.01), lambda=c(0, 0.01, 0.01))
Pt1 <- cbind(1, 2, 3)
## The result of this should approximately 0.01^2/2
print(sphere.tri.area(P, Pt)/(0.01^2/2))
```

```
## Now check that it works for both
P <- rbind(P, P1)
Pt <- rbind(1:3, 4:6)
## Should have two components
print(sphere.tri.area(P, Pt))
```

`spherical.to.polar.area` *Convert lattitude on sphere to radial variable in area-preserving projection*

Description

Project spherical coordinate system (ϕ, λ) to a polar coordinate system (ρ, λ) such that the area of each small region is preserved.

Usage

```
spherical.to.polar.area(phi, R = 1)
```

Arguments

`phi`	Lattitude
`R`	Radius

Details

This requires

$$R^2 \delta\phi \cos\phi \delta\lambda = \rho\delta\rho\delta\lambda$$

. Hence

$$R^2 \int_{-\pi/2}^{\phi} \cos\phi' d\phi' = \int_0^{\rho} \rho' d\rho'$$

. Solving gives $\rho^2/2 = R^2(\sin\phi + 1)$ and hence

$$\rho = R\sqrt{2(\sin\phi + 1)}$$

.

As a check, consider that total area needs to be preserved. If ρ_0 is maximum value of new variable then $A = 2\pi R^2(\sin(\phi_0) + 1) = \pi\rho_0^2$. So $\rho_0 = R\sqrt{2(\sin\phi_0 + 1)}$, which agrees with the formula above.

Value

Coordinate `rho` that has the dimensions of length

Author(s)

David Sterratt

sphericalplot.reconstructedDataset

Spherical plot of reconstructed outline

Description

Draw a spherical plot of datapoints.

Usage

```
## S3 method for class 'reconstructedDataset'
sphericalplot(r,
  datapoints = TRUE, ...)
```

Arguments

r	reconstructedOutline object
datapoints	If TRUE, display data points
...	Other graphics parameters – not used at present

Author(s)

David Sterratt

sphericalplot.reconstructedOutline

Spherical plot of reconstructed outline

Description

Draw a spherical plot of reconstructed outline. This method just draws the mesh.

Usage

```
## S3 method for class 'reconstructedOutline'
sphericalplot(r,
  strain = FALSE, surf = TRUE, ...)
```

Arguments

r	reconstructedOutline object
strain	If TRUE, plot the strain
surf	If TRUE, plot the surface
...	Other graphics parameters – not used at present

Author(s)

David Sterratt

StitchedOutline *Stitch together incisions and tears in an AnnotatedOutline*

Description

This stitches together the incisions and tears by inserting new points in the tears and creating correspondences between new points.

Usage

```
StitchedOutline(a)
```

Arguments

a	AnnotatedOutline object

Value

Rset	the set of points on the rim
i0	the index of the landmark
P	a new set of meshpoints
V0	indicies of the apex of each tear
VF	indicies of the forward vertex of each tear
VB	indicies of the backward vertex of each tear
TFset	list containing indicies of points in each foward tear
TBset	list containing indicies of points in each backward tear
gf	new forward pointer list
gb	new backward pointer list
h	correspondence mapping
hf	correspondence mapping in forward direction for points on boundary
hb	correspondence mapping in backward direction for points on boundary

Author(s)

David Sterratt

`strain.colours` *Generate colours for strain plots*

Description

Generate colours for strain plots

Usage

```
strain.colours(x)
```

Arguments

`x`	Vector of values of log strain

Value

Vector of colours corresponding to strains

Author(s)

David Sterratt

`stretchMesh` *Stretch mesh*

Description

Stretch the mesh in the flat retina to a circular outline

Usage

```
stretchMesh(Cu, L, i.fix, P.fix)
```

Arguments

`Cu`	Edge matrix
`L`	Lengths in flat outline
`i.fix`	Indicies of fixed points
`P.fix`	Coordinates of fixed points

Value

New matrix of 2D point locations

Author(s)

David Sterratt

titrate.reconstructedOutline *Titrate values of phi0*

Description

Try a range of values of phi0s in the reconstruction, recording the energy of the mapping in each case.

Usage

```
titrate.reconstructedOutline(r, alpha = 8, x0 = 0.5,
  byd = 1, len.up = 5, len.down = 20)
```

Arguments

`r`	`ReconstructedOutline` object
`alpha`	Area penalty scaling coefficient
`x0`	Area cutoff coefficient
`byd`	Increments in degrees
`len.up`	How many increments to go up from starting value of `phi0` in `r`.
`len.down`	How many increments to go up from starting value of `phi0` in `r`.

Value

dat Output data frame

Author(s)

David Sterratt

trace.ray *Trace ray through system*

Description

Trace ray through system

Usage

```
trace.ray(r0, S)
```

Arguments

`r0`	Initial ray vector
`S`	System defined by data frame containing left margin of surfaces (A), radii of surfaces (R), cut-offs (C) and refracive indices (n).

Value

5-column matrix with Cartesian coordinates of ray origin in first two columns (`x` and `y`), angles of rays to x-axis in third column (`theta`), index of surface at which intersection occurs (`j`) and angle to x-axis at which intersection occurs on surface (`alpha`).

Author(s)

David Sterratt

`trace.ray.angle` *Trace ray at an angle*

Description

Trace ray at an angle

Usage

```
trace.ray.angle(psi, anp, S)
```

Arguments

`psi`	Angle from axis
`anp`	Nodal point (hopefully anterior nodal point)
`S`	System

Value

Ray - see `trace.ray`

Author(s)

David Sterratt

`transform.image.reconstructedOutline` *Transform an image into the reconstructed space*

Description

Transform an image into the reconstructed space. The four corner coordinates of each pixel are transformed into spherical coordinates and a mask matrix with the same dimensions as `im` is created. This has `TRUE` for pixels that should be displayed and `FALSE` for ones that should not.

Usage

```
transform.image.reconstructedOutline(r)
```

Arguments

`r`	`reconstructedOutline` object

Value

`reconstructedOutline` object with extra elements

`ims`	Coordinates of corners of pixes in spherical coordinates
`immask`	Mask matrix with same dimensions as image `im`

Author(s)

David Sterratt

`tri.area` *Area of triangles on a plane*

Description

Area of triangles on a plane

Usage

```
tri.area(P, Pt)
```

Arguments

`P`	2-column matrix of vertices of triangles
`Pt`	3-column matrix of indicies of rows of `P` giving triangulation

Value

Vectors of areas of triangles

Author(s)

David Sterratt

`tri.area.signed` *"Signed area" of triangles on a plane*

Description

"Signed area" of triangles on a plane

Usage

```
tri.area.signed(P, Pt)
```

Arguments

`P`	2-column matrix of vertices of triangles
`Pt`	3-column matrix of indicies of rows of `P` giving triangulation

Value

Vectors of signed areas of triangles. Positive sign indicates points are anticlockwise direction; negative indicates clockwise.

Author(s)

David Sterratt

`TriangulatedOutline` *Triangulate outline*

Description

Create a triangulation of the `Outline` object `o`. The minimum number of triangles in the triangulation is specified by `n`.

Usage

```
TriangulatedOutline(o, n = 200,
  suppress.external.steiner = FALSE)
```

Arguments

`o`	`Outline` object
`n`	Minimum number of points in the triangulation
`suppress.external.steiner`	If `TRUE` prevent the addition of points in the outline. This happens to maintain triangle quality.

Value

A `triangulatedOutline` object containing the following fields:

`P`	The set of new points, with the existing points at the start
`T`	The triangulation
`Cu`	Unique set of M connections, as M*2 matrix
`h`	Correspondances mapping
`A`	Array containing area of each triangle
`L`	Length of each connection
`A.signed`	Signed area of each triangle
`A.tot`	Total area of outline
`gf`	Forward pointers
`gb`	Backward pointers
`S`	Segments (from `triangulate`)
`E`	Edges (from `triangulate`)
`EB`	Edge boundaries (from `triangulate`)

Author(s)

David Sterratt

`vecnorm` *Vector norm*

Description

Vector norm

Usage

```
vecnorm(X)
```

Arguments

`X`	Vector or matrix.

Value

If a vector, returns the 2-norm of the vector. If a matrix, returns the 2-norm of each row of the matrix

Author(s)

David Sterratt

`whichTear` *Return index of tear*

Description

Return index of tear in an AnnotatedOutline in which a point appears

Usage

```
whichTear(o, pid)
```

Arguments

`o`	`AnnotatedOutline` object
`pid`	ID of point

Value

ID of tear

Author(s)

David Sterratt

Index

addClass, 4
addTear, 5
AnnotatedOutline, 6, *20*, *24*, *27*, *68*, *82*, *84*, *92*
azel.to.sphere.colattitude, 6
azimuthal.conformal, 7
azimuthal.equalarea, 8, *62*, *63*
azimuthal.equidistant, 8

bary.to.sphere.cart, 9

central.angle, 10, *46*
checkDatadir, 10
checkTears, 11
circle, 11
compute.intersections.sphere, 12
compute.kernel.estimate, 13, *37*, *38*
computeTearRelationships, 14
create.polar.cart.grid, 14
csv.read.dataset, 15, *80*

Dataset, 16, *23*, *24*, *35*
dE, 16
draw.arc, 17
draw.system, 18

E, 18
Ecart, 19
ensureFixedPointInRim, 20

f, 21, *29*
Fcart, 21
fire, 22, *86*
flatplot, 23
flatplot.annotatedOutline, 24
flatplot.dataset, 24
flatplot.outline, 25
flatplot.reconstructedOutline, 25
flatplot.retinalDataset, 26
flatplot.stitchedOutline, 27
flatplot.triangulatedOutline, 27
flipped.triangles, 28
flipped.triangles.cart, 28
fp, 29

getDss, 30
getDss.reconstructedDataset, 30
getDss.retinalReconstructedDataset, 31
getDssHullarea, 31
getDssMean, 32
getDssMean.reconstructedDataset, 32
getDssMean.retinalReconstructedDataset, 33
getGss, 33
getGss.reconstructedDataset, 34
getGss.retinalReconstructedDataset, 34
getIDs, *24*, 35, *62*
getIDs.dataset, 35
getIDs.reconstructedDataset, 36
getIms, 36
getIms.reconstructedOutline, 37
getKDE, 37
getKR, 38
getSss, 38
getSss.reconstructedDataset, 39
getSss.retinalReconstructedDataset, 39
getStrains, 40
getTear, 40
getTss.reconstructedOutline, 41
getTss.retinalReconstructedOutline, 41

identity.transform, 42
idt.read.dataset, 42, *80*
ijroi.read.dataset, 43, *80*
incident.to.lens.angle, 44
invert.sphere, 45
invert.sphere.to.hemisphere, 45

karcher.mean.sphere, 46
kde.compute.concentration, 47
kde.fhat, *13*, 47
kde.fhat.cart, 48
kde.L, 48
kr.compute.concentration, 49

kr.sscv, 49
kr.yhat, *13*, 50
kr.yhat.cart, 50

labelTearPoints, 51
line.line.intersection, 51
list.datasets, 52
lvsLplot.reconstructedOutline, 53

mergePointsEdges, 53, *64*

name.list, 54
nameLandmark.dataset, 55
nameLandmark.retinalDataset, 55
new.ray, 56
normalise.angle, 56

optimiseMapping, 57
orthographic, 58
Outline, *23*, *25*, 58, *97*

panlabel, 59
plot.IJROI, 59
polar.cart.to.sphere.spherical, 60, *88*
polartext, 61
projection, 61, *61*
projection.reconstructedDataset, *61*, 62
projection.reconstructedOutline, *61*, 63
projectToSphere, 64

ray.arc.intersection, 64
Rcart, 65
read.datapoints, *15*, *43*, 66
read.ijroi, 66
ReconstructedDataset, *30–34*, *36–38*, *62*, 67, *67*, *79*, *83*
ReconstructedOutline, *25, 26*, *36, 37*, *40*, *61*, *63*, 68, *79*, *82, 83*, *94*
remove.identical.consecutive.rows, 69
remove.intersections, 70
removeTear, 70
RetinalDataset, *43*, 71, *80*, *82, 83*
RetinalReconstructedDataset, *30–34*, *38, 39*, 71, *71*, *82*
RetinalReconstructedOutline, *36*, *41*, 72, *82*
retistruct.batch, 72
retistruct.batch.analyse.summaries, 73
retistruct.batch.analyse.summary, 74
retistruct.batch.export.matlab, 74
retistruct.batch.figures, 75
retistruct.batch.plot.ods, 75
retistruct.batch.summary, *75*, 76
retistruct.check.markup, 76
retistruct.cli, 77
retistruct.cli.figure, 77
retistruct.cli.process, *77*, 78
retistruct.export.matlab, 79
retistruct.potential.od, 79
retistruct.read.dataset, 80, *81*
retistruct.read.markup, 80
retistruct.read.recdata, 81
retistruct.reconstruct, 82
retistruct.save.markup, 82
retistruct.save.recdata, 83
rotate.axis, 83

setFixedPoint, 84
simplify.outline, 85
sinusoidal, *62, 63*, 85
solveMappingCart, 86
sphere.cart.to.sphere.spherical, 87
sphere.spherical.to.polar.cart, *60*, 88
sphere.spherical.to.sphere.cart, 88
sphere.tri.area, 89
spherical.to.polar.area, 90
sphericalplot.reconstructedDataset, 91
sphericalplot.reconstructedOutline, 91
StitchedOutline, *27*, 92
strain.colours, 93
stretchMesh, *64*, 93

titrate.reconstructedOutline, *78*, 94
trace.ray, 94, *95*
trace.ray.angle, 95
transform.image.reconstructedOutline, 95
tri.area, 96
tri.area.signed, 97
triangulate, *98*
TriangulatedOutline, *27*, 97

vecnorm, 98

`whichTear`, 99
