# Supplementary material for: Standard Anatomical and Visual Space for the Mouse Retina: Computational Reconstruction and Transformation of Flattened Retinae with the Retistruct Package
Source: PLoS Comput Biol. 2013 Feb 28;9(2):e1002921. doi: 10.1371/journal.pcbi.1002921 (PMC3585388; doi:10.1371/journal.pcbi.1002921)
Supplement: Text S1 — Supplemental information including an extended Discussion, and Materials and Methods containing details of experiments and detailed description of the reconstruction algorithm and analysis of reconstructed data. (PDF) [file pcbi.1002921.s002.pdf]

Supplemental information for "Standard anatomical and visual space for the mouse retina: computational reconstruction and transformation of flattened retinae with the Retistruct package"

David C. Sterratt[1*#], Daniel Lyngholm[2#], David J. Willshaw[1], Ian D. Thompson[2]
1 Institute for Adaptive and Neural Computation, School of Informatics, University of Edinburgh, Edinburgh, Scotland, UK
2 MRC Centre for Developmental Neurobiology, King's College London, London, UK
*E-mail: david.c.sterratt@ed.ac.uk
#These authors contributed equally to this work

Supplemental Discussion

Assessment of the method

As far as we are aware, a computational algorithm for reconstructing retinal flat-mounts into standard space and then transforming these retinal coordinates into visuotopic coordinates has never been described before. Our validation studies suggest that the method is able to estimate the original location of a point on a flattened retina to within 8° of arc, which is equivalent to 3.6% of the nasotemporal axis. The ability to reconstruct retinae allows for the analysis of populations of labels that would otherwise be split across the cuts or tears of the flat-mount preparation. It also allows retinae from different eyes to be mapped onto a standard retina, enabling cross-eye comparisons to be made. The ability to map images into standard retinal space is a useful method of visualisation, as are the various map projections from standard retinal space. As part of the software package that implements the algorithm, we have provided routines to analyse point data on the sphere.

It is important to understand the limitations of the method and how they might be mitigated. Firstly, the algorithm can only be as good as the experimental error of dissection and fixing fed into it. There is a great deal of variability in dissected retinae: in some it is clear where the vertices of cuts and tears lie and in others is much less so; thus error may be added when the cuts and tears are marked up. The deformation measure gives an indication of the quality of a reconstruction and was higher (worse) for younger retinae (Figure 3B), which are more delicate and harder to dissect cleanly. A high deformation measure indicates that a reconstruction should be treated with caution. In retinae where the locations of the tears and cuts are less clear, trying an alternative marking up can reduce the deformation.

Secondly, the physical model used by the algorithm contains simplifying assumptions. The retinal tissue is modelled as a mesh of masses connected by springs. This basic model is used in modelling deformable surfaces in computer animation and clothes design [1–3] and has been used in models of soft tissue [4]. This model should ensure that neighbourhood relations are preserved – nodes close in the 2D structure should still be close in the 3D structure – and incorporates the notion of an elastic material. However, it does not incorporate an explicit representation of tensile and shear forces.

A more physically-principled method of modelling deformation of objects is offered by the finite element method (FEM), in which the stress and strain of each triangular element in the mesh is derived as a function of the deformation of the points of the element [5]. The stress and strain matrices represent shearing within the material, and materials with varying degrees of compressibility (as quantified by Poisson's ratio) can be modelled. The FEM is used widely to describe properties of soft tissue in simulations of surgery [6]. The FEM could be applied fairly straightforwardly to the forward problem of how a retina with known cuts deforms during flattening. However, it is not possible to apply the FEM directly to the problem of mapping the flattened retina onto the intact retina. This would require knowledge of the stresses and strains in the flattened retina, whereas the only information available is the outline of the retina and the location of the optic disc.

Finally, and less crucially, the meshing algorithm is sensitive to neighbouring points on the outline

being very close together. This leads to a large number of very small triangles being constructed, which can lead to a tangle of many flipped triangles in the energy minimisation phase and, ultimately, small areas of high strain in the reconstructed outline. Suitable prepossessing of outlines to remove very short edges can eliminate this problem.

Possible extensions

In terms of the reconstruction algorithm, it might be possible to automate the mark-up procedure. This would be straightforward for cleanly dissected retinae, but would be difficult for retinae with irregular cuts and tears. Furthermore marking up takes at most a few minutes using the interface supplied with the software. The other piece of information required by the algorithm is the rim angle, which is currently determined by the age of the tissue (Table 1). It would be possible to infer this automatically (Figure 3D) but we decided that the extra complexity did not warrant doing this.

Materials and Methods

Animals

All animals used in this study are on a C57BL/6J background (Charles River, UK). All procedures were performed in accordance with the European Communities Council Directive of 24 November 1986 (86/609/EEC) under the Animals (Scientific Procedures) Act 1986. For surgery, adult animals were given an injection of Vetergesic (0.1 mg/kg) and anaesthetised either with Isoflurane (induction at 4% and maintenance at 2%) or ketamine/xylazine (3 ml/kg of stock solution: 33 mg/ml, Ketamine and 3 mg/ml, Xylazine). Animals were then secured with ear-bars in a custom-made frame and maintained at 37°C throughout surgery. For injections into the dLGN, a skull flap was drilled and either unilateral or bilateral cortical aspiration was performed to reveal the thalamus. Subsequently, either fluorescein-conjugated dextran (Fluoro-Emerald) or tetramethylrhodamine-conjugated dextran (Fluoro-Ruby) (5% w/v; 10,000MW; D1820, D1817; Invitrogen) was pressure-injected through glass pipettes with a 120 µm internal diameter and tip diameters 30–100 µm. Multiple injections were made into the thalamus; total volumes into one thalamus ranged between 50 nl and 250 nl. For injections into the superior colliculus, a skull flap was similarly drilled and unilateral cortical aspiration was performed to reveal the entire superior colliculus. Subsequently, 5–10 nl of Red and Green latex microspheres (RetroBeads; Lumafluor) were pressure-injected into the upper 200-300 µm of the superior colliculus using similar pipettes as above, but with 30–50 µm tip diameters. Following surgery, the cavity from aspiration was filled with absorbable gelatine sponge, the skull-flap replaced and the skin sutured. Animals were allowed to recover and left for 24–48 hours after which they were terminally anaesthetised by intraperitoneal injection of pentobarbital sodium (300 mg/kg) and perfused transcardially with phosphate buffered saline at 4°C followed by paraformaldehyde (4% w/v) at 4°C.

Retinal dissections

With the eye still in the head, a microknife cut was made in nasal cornea at the level of the middle of the nictitating membrane. This allowed a subsequent orienting cut to be made in nasal retina. The eye was removed and dissected using a Sylgard-filled Petri dish and insect-pins. The front of the eye was removed just anterior to the corneo-scleral junction and the lens removed. Fine scissors were used to make a long cut through the sclera, choroid and retina from the nasal orienting cut towards the optic disc. Shorter relaxing cuts were made at approximately 90° or 120° intervals, depending on the age of the animal. The retina was freed from the choroid and cut free at the optic disc. It was then transferred to a Poly-L-Lysine coated microscope slide, mounted ganglion cell layer up and cover-slipped using Fluoromount (Sigma). Retinae were imaged using fluorescence microscopy.

To investigate the orientation of the eye, the insertions of the superior, inferior and lateral rectus muscles were marked onto the retina by passing a needle coated with Fluoro-Emerald or Fluoro-Ruby (5% w/v) through the sclera into the retina at the centre of the muscle-insertion prior to dissecting the retina. The staining for shortwave opsin was done using a polyclonal antibody against S-opsin (1:200; AB5407; Millipore) with a Fluorescein-conjugated secondary antibody (1:1000; AP123F; Millipore).

Reconstruction algorithm

The reconstruction algorithm described here has been implemented in R [7] and can be downloaded as an R package from http://retistruct.r-forge.r-project.org/. It has been tested on GNU/Linux, but should also work in MacOS and Windows. The user manual, available from the same site, contains details of the two main data formats Retistruct can process. These are either in the form of coordinates of data points and retinal outline from an in-house camera-lucida setup, or in the form of bitmap images with an outline marked up in ImageJ ROI format [8]. In this paper, retinae were imaged using an Axiophot2 microscope (Zeiss) and processed to find cell locations using ImageJ. Retinal ganglion cell locations in retinae used for Figures 1, 2 and 5, however, were plotted using an in-house camera-lucida setup. For the latter, retinae were either sampled fully or partially (one in nine 150 µm square boxes).

The steps of the reconstruction algorithm are illustrated in Figure 1. The raw data (Figure 1A) consists of the sequence of points making up the outline, sets of data points and sequences of connected points defining landmarks, such as the optic disc. The reconstruction process then proceeds as follows:

1. The location of one of the nasal position and all the incisions and tears in the outline are marked up by an expert (Figure 1B) using the custom-built graphical user interface. Each tear is defined by a central point, referred to as the apex, and two outer points, referred to as vertices. Tears within tears or cuts can be marked up.

2. The retinal outline is triangulated using the conforming Delaunay triangulation algorithm provided by the Triangle package [9] such that there are at least 500 triangles in the outline (grey lines in Figure 1C).

3. The tears and cuts are stitched automatically (cyan lines in Figure 1D). To do this, firstly the length of each side of every tear is computed. The fractional distance of each point in each tear is then defined as the distance along the tear of that point from the apex divided by the total length of that side of the tear. For each point on one side of a tear a corresponding point is inserted at the same fractional distance along the opposing side. Tears within tears are dealt with by excluding the child tear when computing the fractional distance. At the end of the procedure there is a set of correspondences between two or, in the case of the vertices of a child tear, three points.

4. There is then an extra round of triangulation to incorporate the points that have been inserted into cuts and tears.

5. The points within each set of correspondences are merged and allocated positions on the 2D surface.

6. The triangulation points are then projected onto a sphere curtailed at latitude of φ_0 (Figure 1D). Note that whilst we present the coordinates of the standard retina in terms of colatitude and longitude, all internal calculations use latitude and longitude. Colatitude is converted to latitude by subtracting 90°. The latitude is estimated on the basis of measurements from intact retinae of animals of the same age as the retina under reconstruction. The radius R of the sphere is determined by the area of the flattened retina and φ_0. Points on the rim of flattened retina are fixed to the rim of the curtailed sphere.

7. The optimal projection onto the curtailed sphere (Figure 1E) is inferred by shifting the locations of the vertices on the spherical surface so as to minimise the energy measure, E, which comprises the sum of normalised squared differences between lengths of corresponding connections in flattened and spherical retina (e_L^2, see Equation 1) and a penalty term that prevents the triangles from flipping over:
$$E = \frac{1}{2|\mathcal{C}|\overline{L}} \sum_{i \in \mathcal{C}} \frac{(l_i - L_i)^2}{L_i} + \beta \sum_{j \in \mathcal{T}} \left(A_j / \overline{A}\right)^{\nu} f\left(a_j / A_j\right) \tag{1}$$
where L_i and l_i are lengths of corresponding edges $i \in \mathcal{C}$ in the flattened and spherical retina respectively, $\overline{L}$ is the mean length of an edge, $|\mathcal{C}|$ is the number of edges, f is a penalty function to be defined below, and A_j and a_j are signed areas of corresponding triangles $j \in \mathcal{T}$ in the flattened and spherical retina and $\overline{A}$ is the mean of A_j. The parameter β controls the relative contribution of the area penalty, and ν controls how much larger triangles are penalised than smaller ones; they are set as described below. The signed area a_j is positive for triangles in correct orientation, but negative for flipped triangles. The penalty function f is a piecewise, smooth function that increases with negative arguments:
$$f(x) = \begin{cases} -\left(x - x_0\right)/2 & x < 0 \\ \frac{1}{2x_0}\left(x - x_0\right)^2 & 0 < x < x_0 \\ 0 & x > x_0 \end{cases} \tag{2}$$
The parameter x_0 is set at 0.5. There is thus no penalty unless triangles have been squashed to less than 50% of their size in the flattened retina. The length of each edge l_i is computed from the formula for the central angle between its vertices:
$$l\left(\varphi_1, \lambda_1, \varphi_2, \lambda_2\right) = R \arccos\left(\cos\varphi_1 \cos\varphi_2 \cos\left(\lambda_1 - \lambda_2\right) + \sin\varphi_1 \sin\varphi_2\right) \tag{3}$$
where φ_1 and φ_2 are the latitudes of the vertices and λ_1 and λ_2 are the longitudes. The derivatives of E with respect to φ_i and λ_i are computed and used to minimise E. Optimisation proceeds by: (i) turning off the area penalty $\beta = 0$ and using the FIRE structural relaxation algorithm [10]; (ii) starting from this configuration, setting $\beta = 8$ and $\nu = 1$ and again using FIRE to minimise the energy, which removes a number of the flipped triangles, dealing preferentially with the biggest; and (iii) minimising using the BFGS quasi-Newton method (as implemented in the R optim function) with $\beta = 8$ and $\nu = 0.5$ to refine the optimisation. This procedure was arrived at after trials on a corpus of over 200 marked-up retinae.

8. The locations of data points and landmarks on the sphere are determined (Figure 1F). To do this, for each data point the barycentric coordinates within its containing triangle in the flat representation are determined. The location of the point on the sphere is then found by projecting the point to the same set of barycentric coordinates in the corresponding triangle on the sphere. From this location in 3D space, represented in Cartesian coordinates, the spherical coordinates of the point are determined by projection of a line from the centre of the sphere through the point to the line's intersection with the sphere. This allows plotting of points in a polar representation. The procedure can be used in reverse to infer the locations of lines of latitude and longitude in the flat retina.

The procedure of reconstructing a retina takes around 1 minute (range 20 seconds to 11 minutes) on a Dell Optiplex 990 with an i7-2600 CPU running Scientific Linux 5.

Projections used to display of reconstructed data

The data on the sphere can be projected onto a polar or azimuthal equidistant plot in the plane with coordinates (ρ, λ) where the radius $\rho = \pi/2 + \varphi$. Alternatively the data can be plotted in an azimuthal equal area (Lambert) projection [11], in which area is preserved in the sense that equal areas on the

sphere project to equal areas on the plane of projection, achieved by setting $\rho = \sqrt{2(1+\sin\varphi)}$. Finally the data can be plotted in an azimuthal conformal (Wulff) projection in which angles are preserved; this is achieved by setting $\rho = \tan(\pi/4 + \varphi/2)$.

For data transformed into visuotopic space (see later), we used the sinusoidal orthographic projections. The sinusoidal projection, which projects the entire globe onto the plane and preserves area, is given by $x = (\lambda - \lambda_0)\cos\varphi, y = \varphi$, where λ_0 is the longitude at the centre of the projection [12]. The orthographic projection, which gives a perspective view of one side of the globe, is given by $x = \cos\varphi\sin(\lambda - \lambda_0), y = \cos\varphi_0\sin\varphi - \sin\varphi_0\cos\varphi\cos(\lambda - \lambda_0)$, where the projection is centred on (φ_0, λ_0) [13].

By convention, the polar plots are viewed as though the animal is facing towards the observer. This means that when plotting a retina from a right eye, the nasal pole on the right and N, D, T, V are in anticlockwise order; for a retina from a left eye, nasal is on the left and N, D, T, V are in clockwise order. The longitude of a point was defined so that 0° is always at the right of the plot and 90° at top. This means that for a right eye the poles correspond to the longitudes as follows: N, 0°; D, 90°; T, 180°; V, 270°. For a left eye the longitudes of the nasal and temporal poles are interchanged: N, 180°; D, 90°, T, 0°; V, 270°.

Analysis of reconstructed data

Mean location of data points The Karcher mean of a set of points on the sphere [14,15] is defined as the point $(\overline{\varphi}, \overline{\lambda})$ that has a minimal sum of squared distances to the set of points (φ_i, λ_i) :

$$(\overline{\varphi}, \overline{\lambda}) = \arg\,\min_{(\varphi,\lambda)} \sum_{i=1}^{N} l^2(\varphi, \lambda, \varphi_i, \lambda_i) \quad (4)$$

where the function l is defined in Equation 5.

Kernel density estimates We use Diggle and Fisher's method for producing kernel density plots of data points lying on a sphere [16]. The underlying kernel function is the Fisherian density [17]:

$$F(\varphi, \lambda, \varphi_0, \lambda_0; \kappa) = \frac{\kappa}{4\pi\cosh\kappa}\exp(\kappa\cos l(\varphi, \lambda, \varphi_0, \lambda_0)) = \frac{\kappa}{4\pi\cosh\kappa}\exp(\kappa\vec{r}\cdot\vec{r}_0) \quad (5)$$

where κ is the "concentration" parameter and $\vec{r}$ and $\vec{r}_0$ are the Cartesian coordinates of points with spherical coordinates (φ, λ) and (φ_0, λ_0) on the unit sphere. For large κ the Fisherian density is approximately a bivariate Gaussian with variance $\sigma^2 = \kappa^{-1}$ [16]. The density at a location (φ, λ) is defined as:

$$\hat{f}(\varphi, \lambda; \kappa) = \frac{1}{n}\sum_{i=1}^{n} F(\varphi, \lambda, \varphi_i, \lambda_i; \kappa) \quad (6)$$

κ is chosen so as to maximise a cross-validated log-likelihood:

$$L(\kappa) = \sum_{i=1}^{n} \log\{\hat{f}_i(\varphi_i, \lambda_i; \kappa)\} \quad (7)$$

where $\hat{f}_i(\varphi, \lambda; \kappa)$ is the kernel density estimate calculated from all but the ith data point.

Kernel regression estimates In kernel regression, we have values y_i at each data point (φ_i, λ_i). We adapt the Nadaraya-Watson estimator [18,19] to the Fisher density and the spherical coordinates:

$$\hat{y}(\varphi, \lambda; \kappa) = \frac{\sum_{i=1}^{n} F(\varphi, \lambda, \varphi_i, \lambda_i; \kappa) y_i}{\sum_{i=1}^{n} F(\varphi, \lambda, \varphi_i, \lambda_i; \kappa)} \quad (8)$$

The concentration parameter κ is set by minimising the summed squared error:

$$\sum_{i=1}^{n}(y_i - \hat{y}_i(\varphi_i, \lambda_i; \kappa))^2 \quad (9)$$

where $\hat{y}_i(\varphi_i, \lambda_i; \kappa)$ is the kernel regression estimate of y_i calculated from all but the ith data point.

Contour analysis The kernel estimates can be represented using a contour plot. This is produced by the standard method [11] of laying a 100 by 100 Cartesian grid over the azimuthal area-preserving (Lambert) projection, finding the spherical coordinates corresponding of each point on the grid, and then computing the density estimate at each point. A standard contouring algorithm is then used to plot the contours. There are a number of methods for determining contour heights [11]. We chose to define them in terms of the amount of probability mass that they exclude; e.g. the 5% contour excludes 5% of the probability mass. The area contained within each contour can be determined simply by finding the area within the contour in the area-preserving (Lambert) projection [11].

Mapping of retina onto visual space

In previous work of the mapping of visual space on the superior colliculus [20], visual space has been described in terms of azimuth θ and elevation α with respect to the long axis of the mouse. Visual space is mapped onto the retina via the optical system of the eye. This mapping depends on the angle that a ray makes with the optic axis, which we define as being oriented at azimuth θ_0 and elevation α_0.

In order to map the eye onto visual space, we consider a reference frame in which the z-axis is vertical, the x-axis is pointing forwards along the long axis of the mouse and the y-axis is pointing rightwards, as viewed by an observer facing the animal. Notionally, the optic axis of the eye starts off vertical, and a sequence of rotations is undertaken in order to move the eye to its correct orientation. First the projection of rays from the retina to visual space is determined using the approximate optics defined in the main text. Second, the coordinates of points are converted to Cartesian coordinates. Third, the coordinates are rotated about the x-axis by $-90 + \alpha_0$ degrees. This ensures that the optic axis is oriented at the elevation α above the horizontal, but the direction of its azimuth will be $-90°$. The points on the eye are then rotated about the original z-axis by $90 + \theta_0$ degrees giving the optic axis an azimuth of θ_0, where positive values are to the right, as viewed from a point on the positive x-axis. Finally the Cartesian coordinates are transformed back to spherical coordinates in the original reference frame.

References

1. Fan J, Wang Q, Chen SF, Yuen MMF, Chan CC (1998) A spring-mass model-based approach for warping cloth patterns on 3D objects. J Visual Comp Animat 9: 215-227.

2. McCartney J, Hinds BK, Seow BL (1999) The flattening of triangulated surfaces incorporating darts and gussets. Comput Aided Design 31: 249-260.

3. Wang CCL, Smith SSF, Yuen MMF (2002) Surface flattening based on energy model. Comput Aided Design 34: 823-833.

4. Škrinjar OM, Duncan JS (1999) Real time 3D brain shift compensation. In: Kuba A, Šáamal M, Todd-Pokropek A, editors, Information Processing in Medical Imaging, Springer, volume 1613 of *Lecture Notes in Computer Science*. pp. 42-55. doi:10.1007/3-540-48714-X_4.

5. Zienkiewicz OC, Taylor RL (2000) The Finite Element Method. Oxford: Butterworth-Heinemann, fifth edition.

6. Carter TJ, Sermesant M, Cash DM, Barratt DC, Tanner C, et al. (2005) Application of soft tissue modelling to image-guided surgery. Medical Engineering & Physics 27: 893-909.

7. R Development Core Team (2011) R: A Language and Environment for Statistical Computing. R Foundation for Statistical Computing, Vienna, Austria. URL `http://www.R-project.org/`. ISBN 3-900051-07-0.

8. Rasband WS (1997-2012). ImageJ, National Institutes of Health, Bethesda, Maryland. URL `http://imagej.nih.gov/ij/`.

9. Shewchuk JR (1996) Triangle: Engineering a 2D quality mesh generator and Delaunay triangulator. In: Lin MC, Manocha D, editors, Applied Computational Geometry: Towards Geometric Engineering, Springer-Verlag, volume 1148 of *Lecture Notes in Computer Science*. pp. 203-222. From the First ACM Workshop on Applied Computational Geometry.

10. Bitzek E, Koskinen P, Gähler F, Moseler M, Gumbsch P (2006) Structural relaxation made simple. Phys Rev Lett 97: 170201.

11. Fisher NI, Lewis T, Embleton BJJ (1987) Statistical analysis of spherical data. Cambridge, UK: Cambridge University Press.

12. Weisstein EW. Sinusoidal projection. From MathWorld–A Wolfram Web Resource. URL `http://mathworld.wolfram.com/SinusoidalProjection.html`.

13. Weisstein EW. Orthographic projection. From MathWorld–A Wolfram Web Resource. URL `http://mathworld.wolfram.com/OrthographicProjection.html`.

14. Karcher H (1977) Riemannian center of mass and mollifier smoothing. Commun Pur Appl Math 30: 509-541.

15. Heo G, Small CG (2006) Form representations and means for landmarks: A survey and comparative study. Comput Vis Image Und 102: 188-203.

16. Diggle PJ, Fisher NI (1985) SPHERE: A contouring program for spherical data. Comput Geosci 11: 725-766.

17. Fisher R (1953) Dispersion on a sphere. Proc R Soc Lond, A 217: 295-305.

18. Nadaraya EA (1964) On estimating regression. Theory Probab Appl 9: 141-142.

19. Watson GS (1964) Smooth regression analysis. Sankhya Ser A 26: 359-372.

20. Dräger UC, Hubel DH (1976) Topography of visual and somatosensory projections to mouse superior colliculus. J Neurophysiol 39: 91-101.
